# So far but so close: the biogeography of soil and plant-associated fungi in one of the most remote landmasses on Earth

**DOI:** 10.1093/ismeco/ycag095

**Published:** 2026-04-14

**Authors:** Constance Bertrand, Françoise Binet, Martino Adamo, Marie-Claire Martin, Roland Marmeisse

**Affiliations:** Univ Rennes, CNRS, ECOBIO [(Ecosystèmes, biodiversité, évolution)]—UMR 6553, F-35000 Rennes, France; UMR ISYEB 7205 CNRS-MNHN-Sorbonne Université-EPHE-UA, Muséum National d'Histoire Naturelle, 57 rue Cuvier, 75005 Paris, France; Univ Rennes, CNRS, ECOBIO [(Ecosystèmes, biodiversité, évolution)]—UMR 6553, F-35000 Rennes, France; DBIOS Università degli Studi di Torino, Orto Botanico di Torino, 25 Viale Pier Andrea Mattioli, 10125 Torino, Italy; Univ Rennes, CNRS, ECOBIO [(Ecosystèmes, biodiversité, évolution)]—UMR 6553, F-35000 Rennes, France; UMR ISYEB 7205 CNRS-MNHN-Sorbonne Université-EPHE-UA, Muséum National d'Histoire Naturelle, 57 rue Cuvier, 75005 Paris, France

**Keywords:** fungal biogeography, endemism, biological invasion, Kerguelen Islands, sub-Antarctic

## Abstract

The Kerguelen Islands, one of the most isolated lands on Earth, represent an ideal open-air laboratory to explore patterns of microbial biogeography under minimal human influence. Fellfields, in particular, are near pristine habitats dominated by endemic plant species with few introduced ones. Using metabarcoding, we characterized 70 bulk soil and 70 root-associated fungal communities from two native plant species and one introduced one, in four distant fellfield sites. Comparative analyses of Kerguelen fungal sequences with global reference databases (GlobalFungi and UNITE) revealed that 60% to 76% of the recovered operational taxonomic units (OTUs) had a close match at ≥97% sequence identity, which indicates the presence of a majority of species with wide distribution ranges that have already been observed elsewhere on the globe. Although evidence of endemism is difficult to establish, haplotype networks created for OTUs already observed elsewhere in the world illustrate in a number of cases the presence of dominant amplified sequenced variants specific to Kerguelen, suggesting intraspecific endemism. A global analysis of the already known fungal OTUs showed they were predominantly associated to high-latitude and cold environments. A spatial analysis further affiliated known Kerguelen’s fungi to two distinct endemicity zones, one that encompassed Southern South America/Antarctic Peninsula and one in Central and Northern Europe that potentially contributed to alien species that may have invaded this remote archipelago. Our results indicate that the mycoflora of one of the most isolated islands in the world has been shaped by repeated episodes of colonization from different parts of the globe.

## Graphical Abstract

**Graphical Abstract:**
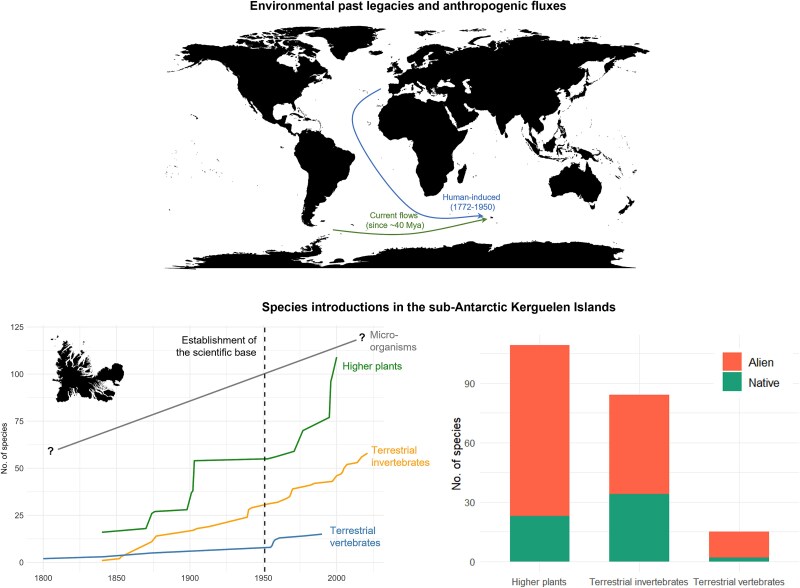

## Introduction

Fungi play key roles in terrestrial ecosystems: their contributions to nutrient cycling [1, 2], soil structure [3], and plant health [4–6] are essential for maintaining ecosystem functioning. However, despite fungi being of great ecological importance, their biogeographical distribution remains debated, partly because, alike other microscopic organisms, the majority of species have not been described yet [7]. Another major point regards whether fungi can exhibit high levels of endemism or whether they are ubiquitously distributed due to their dispersal capabilities. This debate is encapsulated in two contrasting paradigms. One is the Baas-Becking hypothesis, ‘everything is everywhere, but the environment selects’ [8], which emphasizes that fungal taxa must possess traits allowing them to survive and thrive under specific environmental conditions. The second is the idea that dispersal limitation and past historical events create distinct biogeographical patterns alike those of macroorganisms, with endemic lineages restricted to certain regions [9].

Studies carried out at different scales showed that, while some fungal groups show cosmopolitan distributions [10–12], others appear to be restricted to specific regions [13, 14]. Fungal dispersal abilities may explain observed biogeographic patterns, as they vary across taxa and are influenced by traits such as spore production, size, longevity, and dispersal vectors (e.g. wind, water, or animals) [15]. Some fungi produce abundant, airborne spores that are capable of long-distance dispersal, allowing cosmopolitan distributions [16, 17]. In contrast, groups such as Glomeromycota, have large spores and rely mostly on root-to-root contact or soil-bound spores, and therefore are thought to have more restricted ranges and possibly higher potential for regional endemism. However, even within Glomeromycota, evidence for endemism remains limited, even in isolated insular environments [11, 18].

Understanding fungal endemism is especially relevant in isolated systems such as oceanic islands, where geographic isolation may promote the evolution of unique microbial assemblages. In this context, the Kerguelen Islands, a remote sub-Antarctic archipelago located thousands of kilometres away from the nearest continent, offer an ideal system in which to test hypotheses about fungal endemism given its low plant diversity (only 29 native vascular plant species) but high levels of endemism, especially in fellfield habitats [19]. This low plant diversity may constrain fungal diversity [20–23] through reduced niche availability or select for specialized fungal taxa adapted to local conditions.

Just like for plants, long-term geographic isolation and environmental harshness (nutrient-poor soils, harsh wind conditions, and high frequency of freeze–thaw cycles) may have shaped a mycobiota that is not merely a subset of global fungal diversity, but a signature of the islands’ unique ecological history. Preliminary studies integrating the Kerguelen Islands (and other sub-Antarctic islands) have shown that Kerguelen’s soil microbiota differ from those of Antarctic islands [24] and from the Antarctic continent and peninsula [25]. However, these studies have focused exclusively on nutrient-rich coastal soils and have not considered plant-associated communities or a broader range of soil conditions that could be found in oligotrophic habitats for example.

Beyond contemporary isolation, the Kerguelen Islands and low latitude regions share a deeper biogeographical history that may promote regionalism in fungal communities. While some regions, such as southern Patagonia and New Zealand’s sub-Antarctic islands, are remnants of the ancient supercontinent Gondwana [26], the Kerguelen Islands formed later through hotspot volcanic activity that started around 30 million years ago [27]. When Gondwana fragmented, the Kerguelen Plateau became increasingly isolated drifting away from what is now Antarctica [26, 28], yet retained relict flora and potentially fungi with shared evolutionary origins [29, 30]. Ocean and wind circulations, namely the Antarctic Circumpolar Current and the prevailing westerly winds (e.g. the ‘Roaring Forties’ and ‘Furious Fifties’) respectively, have been found to facilitate invasion of terrestrial invertebrates such as insects [31, 32]. Similarly, these currents may facilitate the oceanic or aerial transport of fungal spores across vast distances, connecting isolated sub-Antarctic islands and contributing to the persistence of related sub-Antarctic biota. Finally, human activities have led to a threefold increase in the number of plant species on the islands since their discovery over 250 years ago. The pace of these invasions accelerated following the establishment of the weather station and scientific base in 1950 [19]. It is therefore likely that microbial groups, including fungi, have also been inadvertently transported and are now established on the islands [33, 34].

This combined influence of historical geographic isolation and human-mediated introductions suggests that fungal diversity on Kerguelen may not be globally ubiquitous, but shaped by regional evolutionary constraints, ecological processes and also by invasion dynamics. In this study, we characterized the soil and plant root-associated fungal communities in four distantly-located fellfield sites of the Kerguelen Islands and compare them to global datasets to test for signatures of endemism and global dispersal. Similarly to plants, we expected the Kerguelen Islands to harbour low fungal diversity with high levels of endemic or region-specific taxa, due to their past isolation and insular context. We also expected alien plants to harbour more cosmopolitan or widely distributed taxa than native plant species. Finally, we hypothesized that Kerguelen fungal communities share similar compositional profiles to other communities from harsh environments (e.g. high-latitude, high-elevation).

## Materials and methods

### Environmental context of the Kerguelen Islands

**Figure 1:**
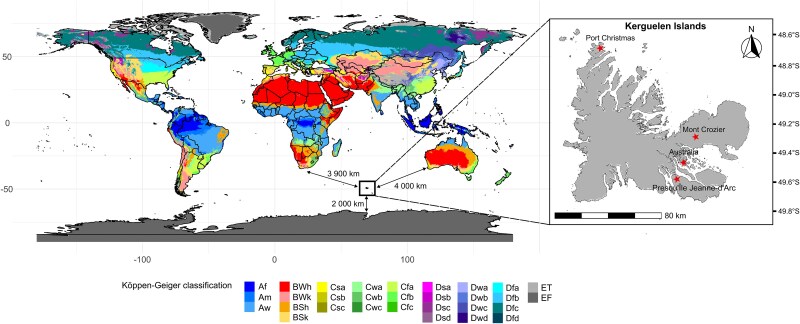
Location of the sub-Antarctic Kerguelen Islands and of the soil and plant sampling (red stars). The background colours represent Köppen–Geiger climate classes (calculated for the 1991–2020 period) according to Beck *et al.* [77]. The Kerguelen archipelago falls within the ET (tundra) climate class (light grey colour).

The sub-Antarctic Kerguelen Islands are a remote archipelago located in the Southern Indian Ocean, ~2000 km from Antarctica and 4000 km from Australia or Africa (Fig. 1). The Kerguelen Islands are characterized by a cold oceanic climate (mean for the 2000–2024 period; annual temperatures of 5.3°C, minimum annual temperatures of 2°C, maximum annual temperatures of 8.6°C, annual precipitation of 703 mm at the Port-aux-Français scientific base; 49°21′S, 70°13E; Météo-France data), often described as cold, buffered and tundra-like, with low annual temperature variation and persistent strong winds. These harsh conditions have constrained the establishment of organisms, resulting in a species-poor native flora and fauna.

Despite its isolation, the Kerguelen Archipelago has undergone progressive ecological shifts ever since its discovery more than 250 years ago, primarily due to 19th century European sealing and whaling activities, followed by farming settlements in the beginning of the 20th century and finally by current scientific and military activities since the establishment of the base in 1951. These successive anthropogenic activities led to the introduction of alien plant species (e.g. *Poa annua, Taraxacum sp.*), terrestrial vertebrates (e.g. rabbits, reindeers) and also various terrestrial invertebrates such as insects, some of which have since become invasive, further altering vegetation cover, ecosystem functioning and fungal communities to the detriment of native species [35–38].

### Plant species of interest

Three plant species were studied. The two phylogenetically distant native species, *Poa kerguelensis* Steud. (Poaceae) and *Pringlea antiscorbutica* R. Br. (Brassicaceae), are endemic to the Southern Indian Ocean Province, which includes Kerguelen, Marion, Crozet, Prince Edward, and Heard Islands. Due to habitat degradation and homogenization from introduced rabbits and alien grasses, both species are now mostly confined to elevated fellfield habitats. In contrast, *P. annua L. *(Poaceae), an alien grass likely introduced by seal hunting activities in the 19th century [34], is widespread and even acts as a pioneer species on glacial moraines and lower-altitude fellfields [39].

### Fellfield sites and soil and plant sample collection

For each plant species, surrounding bulk soil and plant roots were sampled during the 2022–2023 and 2023–2024 austral summers (from December to February) at four different sites of the archipelago to account for spatial heterogeneity (Fig. 1). At each site and for each plant species, ten plant specimen and their surrounding bulk soil were collected along a two-point altitudinal transect with one plot at low-altitude fellfields and the other one at higher-altitude fellfields to account for different environmental conditions along elevational gradients. For each plant species, the 10 specimens were sampled at a similar phenological stage to limit developmental variability.

*Poa kerguelensis* was present and sampled at all four sites, while *P. annua* and *Pringlea antiscorbutica* were found and sampled at only two sites. Moreover, *P. annua* occurred exclusively in the low-altitude fellfields, since it does not colonize high altitude fellfields. In total, 70 roots and their associated 70 soil samples were paired-collected and analysed separately with respect to their fungal communities.

Topsoil, from 0 to 10 cm depth, was collected with a sterilized gouge in the vicinity of the plant on the basis of five soil sub-samplings pooled as one composite bulk soil sample per plant specimen. Roots were cleaned in two successive baths of distilled water followed by one bath of 70% ethanol, and then stored in 70% ethanol-filled Eppendorf tubes. Additional details about the sampling sites and procedures for plant and soil sampling can be found in Bertrand *et al.* ([40]). Processed soil and root samples were then stored at −20°C until further processing.

### DNA extraction and high-throughput sequencing

Roots were first surface sterilized according to a protocol by Wemheuer and Wemheuer ([41]) and then ground in liquid nitrogen with a mortar and pestle. Aliquots of the final bath of water from the surface-sterilization protocol were used as templates in a polymerase chain reaction (PCR) and no PCR products were detected. DNA extractions were performed according to the manufacturer’s instructions using the Power Soil Pro and Plant Pro kits (Qiagen, France) using 250 mg of fresh soil and 100 mg of fresh root samples, respectively.

Metabarcoding of fungal communities in soils and roots was performed by sequencing the ITS2 region of the ITS rDNA gene cluster using primers FITS9 (5′-GAACGCAGCRAAIIGYGA-3′) and ITS4 (5′-TCCTCCGCTTATTGATATGC-3′) [42], extended by Illumina tails. Libraries were prepared by following Illumina 16S Metagenomic Sequencing Library Preparation protocol in two amplification steps: an initial 35 cycle PCR amplification using the locus-specific PCR primers and a subsequent amplification integrating relevant flow-cell binding domains and unique indices (NexteraXT Index Kit, FC-131-1001/FC-131-1002). PCR amplifications, indexing and sequencing were performed by IGA Technology (Udine, Italy) on a NovaSeq6000 instrument (Illumina, San Diego, CA) using a 2x250 bp paired-end mode.

### Bioinformatics

Sequence pre-processing steps and statistical analyses were carried out with R (v. 4.3.0, R Core Development Team, 2005; www.R-project.org).

Paired-end reads generated on the Illumina platform were pre-processed using the DADA2 workflow [43]. Raw reads were first trimmed to remove primers using cutadapt [44] and reads were quality filtered using the *filterAndtrim* function with the following parameters: reads with ambiguous bases and reads with low quality [with maxEE = c(3,3)] and reads that were shorter than 150 bp were filtered out. Error rates were learned from a subset of reads using *learnErrors*(randomize = TRUE), and amplicon sequence variants (ASVs) were inferred using the *dada* function after dereplication. Forward and reverse reads were merged (*mergePairs* function) allowing no mismatches (maxMismatch = 0), and chimeric sequences were removed using *removeBimeraDenovo*(method = ‘consensus’). Sequence alignments were performed with MAFFT (v. 7.520; [45]) and phylogenetic trees were inferred using FastTree2 (v. 2.1.11; [46]). The 38 231 different ASVs were clustered as operational taxonomic units (OTUs) at a 97% identity threshold with the DECIPHER package [47]. The most abundant sequence of each OTU was selected as the OTU representative sequence. Representative sequences were taxonomically assigned using the *assignTaxonomy* and *addSpecies* functions against the UNITE reference database (version 10.0; [48]; https://unite.ut.ee/) with a minimum bootstrap of 60. All non-fungal OTUs or unassigned OTUs at the Phylum level were removed. OTUs representing less than 0.005% of the total dataset were also filtered out. Additionally, we removed putative contaminants using the microDecon package [49] that makes use of sequences amplified from the extraction blank samples. The final dataset included 3097 fungal OTUs.

### Plant- and non-plant-associated taxa selection criteria

From the total dataset, we defined ‘plant-associated’ taxa as fungal OTUs detected in root samples at a relative abundance greater than 0.01%, and present in at least 50% of individuals per plant species in at least one sampling site. This 50% threshold was implemented to account for the potential host specificity and spatial heterogeneity of fungal taxa across the Kerguelen Archipelago; but also to eliminate non-plant-associated taxa accidentally present in one or two root samples because of e.g. insufficient cleaning of the root. Although they represented only ca.3% of the total OTUs richness (*n* = 85), plant-associated taxa accounted for nearly 95% of the total fungal relative abundance in root samples (Supplementary Fig. S1A).

Taxa thereafter referred to as ‘non-plant-associated’ were defined as all remaining OTUs after filtering out plant-associated ones, provided they were present at a relative abundance greater than 0.01% in any bulk soil sample. These non-plant-associated OTUs (*n* = 496) comprised ~16% of the total OTUs and represented more than 96% of the overall relative abundance in soils (Supplementary Fig. S1B).

OTU representative sequences were blasted against GlobalFungi (v.5.0; [50]) and UNITE (v9.0; [48]). While UNITE is a curated collection of taxonomically annotated fungal ITS sequences obtained from different sources (pure cultures, vouchers, environmental samples) and environments (soil, water, sediments, host-associated), the GlobalFungi database compiles exclusively high-throughput (metabarcoding) environmental sequencing data of essentially terrestrial fungal communities worldwide. For taxonomic annotation and biogeography analyses we retained respectively UNITE and GlobalFungi sequences that displayed a >97% identity over their entire length to Kerguelen’s OTU sequences.

From the GlobalFungi database we extracted several metadata associated to sequences corresponding to OTUs identified in the Kerguelen; geographic coordinates (latitude, longitude), mean annual temperature (MAT) and precipitation, soil pH, soil organic carbon (SOC) content, and OTU’s abundance in the original soil samples. Blast searches and metadata retrieval were conducted in January 2025.

### Phylogenetic analysis

All OTUs (*n* = 87) assigned to the order Helotiales, whether plant associated or not, were aligned to 88 representative Helotiales reference sequences from Bruyant *et al.* [5] using MAFFT (v7; [45]) with the L-INS-I method with default parameters and the ‘leave gappy regions’ option. Gblocks (v0.91b) was then used to select for the sites to construct the tree. The phylogenetic tree was constructed on Seaview (v5; [51]) using the PhyML tree option with the GTR model. Branch-support was assessed by bootstrap (1000 replicates).

### Haplotype networks

To evaluate endemism at the intraspecific level, we constructed haplotype networks for each of the 337 Kerguelen OTUs already present in environmental samples listed in the GlobalFungi database. For a given OTU, haplotypes corresponded to its different ASV sequences (≥97% sequence identity between them) that composed it and that were identified in the Kerguelen and/or the GlobalFungi database. ASV sequences were aligned with the ape R package (v.5.1-8; [52]) and haplotype networks were constructed in PopArt using the TCS network algorithm [53].

### Fungal endemicity analyses

We used VNDM version 3.1 [54] to identify areas of fungal endemism based on the spatial distribution of plant- and non-plant-associated Kerguelen OTUs that were also present in the GlobalFungi database. After dividing the entire world into cells of the same size, the VNDM approach evaluates how well each OTU’s distribution fits within sets of grid cells, assigning scores between 0 and 1 that reflect spatial congruence [55]. The algorithm returns a total endemicity score for each area which corresponds to the summed contribution of OTUs best represented in that region. Consensus areas were then defined using a 50% (shared percent of species) cut-off for OTUs similarity among overlapping endemicity zones.

Because the choice of spatial resolution influences endemicity area delineation, we tested four cell sizes (°latitude × longitude): 1° × 1°, 5° × 5°, 10° × 10° and 15° × 15° to cover the entire globe. The 10° × 10° grid was selected as the most appropriate for our dataset, as it was the best compromise between geographic coverage and spatial clustering of OTUs, capturing coherent large-scale endemicity patterns without over-fragmentation.

We further calculated a ‘ubiquity index’ for each selected OTU based on their presence in the 10 plant biomes as delineated by Loidi *et al*. [56] to which was added an 11th one represented by the Antarctic continent. This index ranged from one in case of presence in a single plant biome to 11 when present in all different biomes. We also calculated a ‘tundra specificity percentage’ calculated as the proportion of sampled points contained in one of the cold tundra biomes and/or the Antarctic continent.

**Figure 2:**
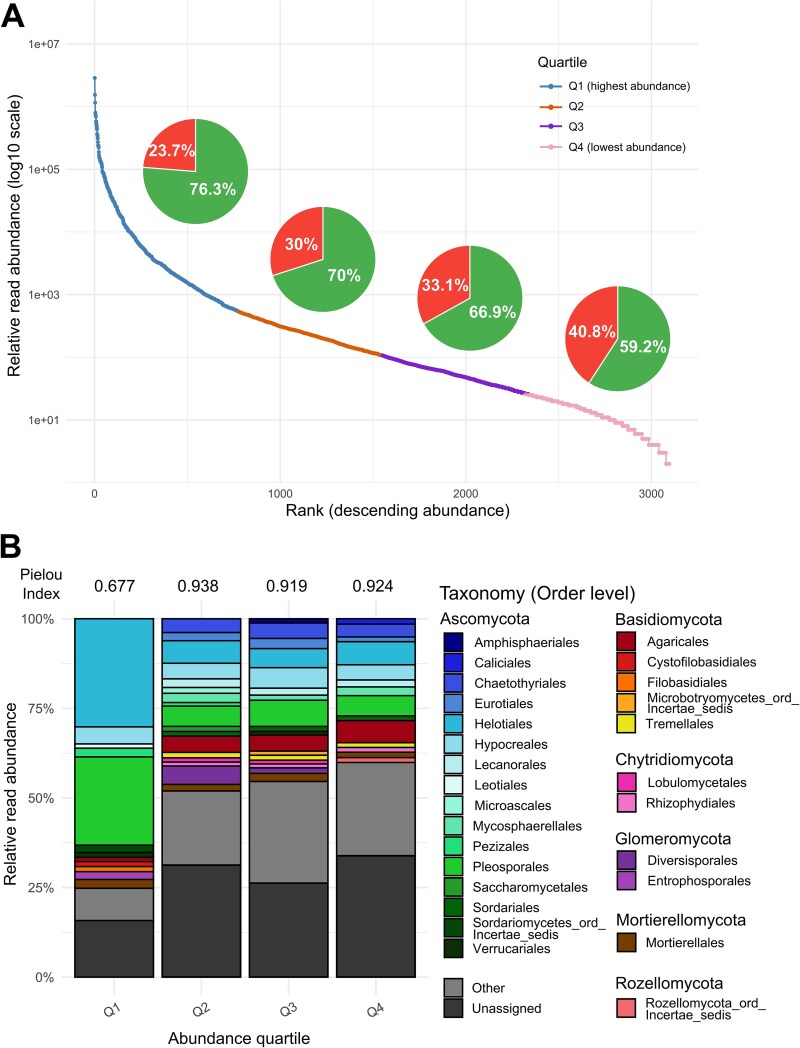
Abundance and taxonomy of fungal OTUs in the whole dataset per quartile (Q1–Q4). Rank-abundance curve with pie charts showing the proportion of OTUs already present (≥97% sequence identity) in UNITE and/or GlobalFungi within each quartile (green) versus unknown ones (<97% identity; red) (A). Taxonomic composition (order level) in each quartile (B). Orders contributing to less than 1% of the relative abundance within a quartile are grouped under the ‘Other’ category. Pielou’s evenness index calculated on read relative abundance at the order level is indicated above each quartile taxonomy bar.

To further characterize the geographic distribution of each OTU present in both the Kerguelen and elsewhere on the globe, we classified OTUs into broad biogeographic categories based on the range of latitudes at which they were detected (see Fig. 2).

### Fungal OTUs abundance pattern in relation to environmental and spatial drivers

In order to evaluate the contribution of environmental variables (MATs and precipitations, SOC content, soil pH) and geographic location (latitude and longitude) on the relative abundance of the known OTUs (*n* = 337, both non-plant- and plant-associated), variance partitioning was performed using the *varpart* function in the vegan package. Spatial structure was derived using Moran’s Eigenvector Maps from sampling coordinates. Statistical significance of each component (environmental and spatial variables) was assessed using partial redundancy analysis and permutational test (*anova.cca*, 1000 permutations).

We modelled OTU abundance using a linear mixed-effects model with the lme4 package [57] to account for potential non-independence due to the primers used, the sample type and the study of origin. Abundance values were log-transformed and all predictors (latitude, MAT, precipitation, soil pH, and SOC) were scaled to mean = 0 and standard deviation = 1. Both linear and quadratic terms were considered for latitude. Normality and homogeneity of variance were evaluated visually.

## Results

### Total OTUs and ASVs and their representation in reference databases of fungal barcode sequences

We identified 3097 distinct fungal OTUs. Among those OTUs, 65% corresponded, at a 97% sequence identity threshold to sequences already present in the public databases GlobalFungi and/or UNITE. The rank-abundance distribution revealed a strong dominance structure, with a clear inflection at the end of the first quartile (Q1), distinct from a long tail of rarer OTUs (Q2–Q4). The proportion of known OTUs (with a ≥97% identity match in either the UNITE or GlobalFungi databases) progressively dropped from almost 80% in Q1 to 60% in Q4 (Fig. 2A). Q1 also differed from the Q2–Q4 quartiles by a skewed taxonomic profile (considering either read or OTU abundance) characterized by a clear dominance of OTUs affiliated to the Helotiales and Pleosporales (Fig. 2B), which both include numerous plant-interacting taxa implicated in plant nutrient uptake in alpine and extreme environments. Quartiles 2 to 4 (Q2–Q4) showed less biased taxonomic profiles compared to Q1, with higher evenness indices (calculated at the order level) (Fig. 2B).

Subsequent analyses were performed on the 581 taxa of the first quartile (Q1) that encompassed 97% of the sequencing reads in order to minimize the contribution of environmental contaminants and of technical artefacts. These dominant OTUs were further categorized as plant-associated (*n* = 85) and non-plant-associated (*n* = 496). Among the remaining dominant OTUs, 84.7% of those identified as plant-associated and 77.4% of non-plant-associated ones were already present in UNITE and/or GlobalFungi.

While initial analyses carried out at the OTU level, most of which group together different ASVs, suggest that a large proportion of observed fungal taxa are not endemic to the Kerguelen, we cannot exclude geographic isolation at the ASV level. Thus, for each of the Kerguelen OTU that corresponded to OTUs already present in the GlobalFungi database (with a >97% sequence identity), we extracted individual ASVs from both the Kerguelen and GlobalFungi datasets to construct as many different haplotype networks (illustrated in Supplementary Fig. S2 for two most abundant OTUs in soils and two most abundant in roots). Only 62 out of these 337 networks (18%) highlighted ASVs specific to the Kerguelen (so-called private alleles in population genetics) (Supplementary Fig. S3 & Table  S1). For 34 out of the 62 OTUs (ca. 55%), the Kerguelen-specific ASVs represented more than 90% of the read abundance (Supplementary Table S1), suggesting endemism at the ASV level.

### Taxonomic overview and Helotiales diversification in the Kerguelen

**Figure 3:**
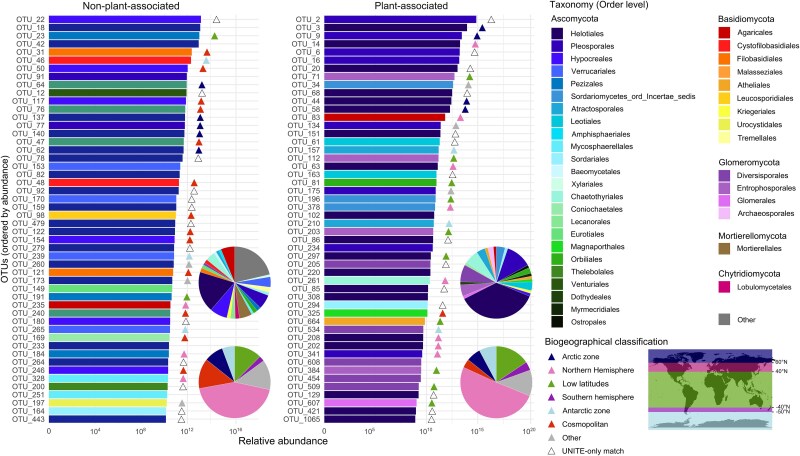
Rank-abundance diagram showing the taxonomy and geographical classification of the 50 most abundant plant-associated and non-plant-associated fungal taxa. The x-axis corresponds to the total number of reads across the whole dataset for each OTU. Coloured triangles indicate a ≥97% sequence identity match against the GlobalFungi database, with an associated biogeographical origin. Empty triangles indicate a ≥97% sequence identity match only in the UNITE database (and <97% in GlobalFungi), meaning that no geographical metadata is available. Taxa with no triangle had a <97% sequence identity matches in both databases. Biogeographical classes were defined purely on the latitudinal distribution of corresponding OTUs in the GlobalFungi database. For each OTU, we calculated the minimum and maximum sampling latitude and assigned it to one of the following classes: *cosmopolitan* (latitudinal range of over 60° and spanning both hemispheres), *Arctic zone* (≥60° N), *Antarctic zone* (≤−50°N), *Northern Hemisphere* (between 40° and 60°N), *Southern Hemisphere* (between −40° and − 50°N), or *low latitudes* (between −40° and 40°N). Taxa not meeting these criteria were classified as *Other*. Pie charts represent the proportion of each order (top) and of each biogeographical class (bottom) across the whole dataset. OTUs representing less than 1% of the whole dataset are grouped in the ‘Other’ category.

We observed distinct taxonomic profiles between plant-associated and non-plant-associated OTUs (illustrated in Fig. 3 for the 50 most abundant OTUs of both categories). While the order Helotiales (Ascomycota) was the most abundant in both groups, plant-associated OTUs were enriched in members of order Pleosporales (Ascomycota) and phylum Glomeromycota, while non-plant-associated OTUs were enriched in taxa belonging to the order Hypocreales (Ascomycota) and phylum Basidiomycota.

**Figure 4:**
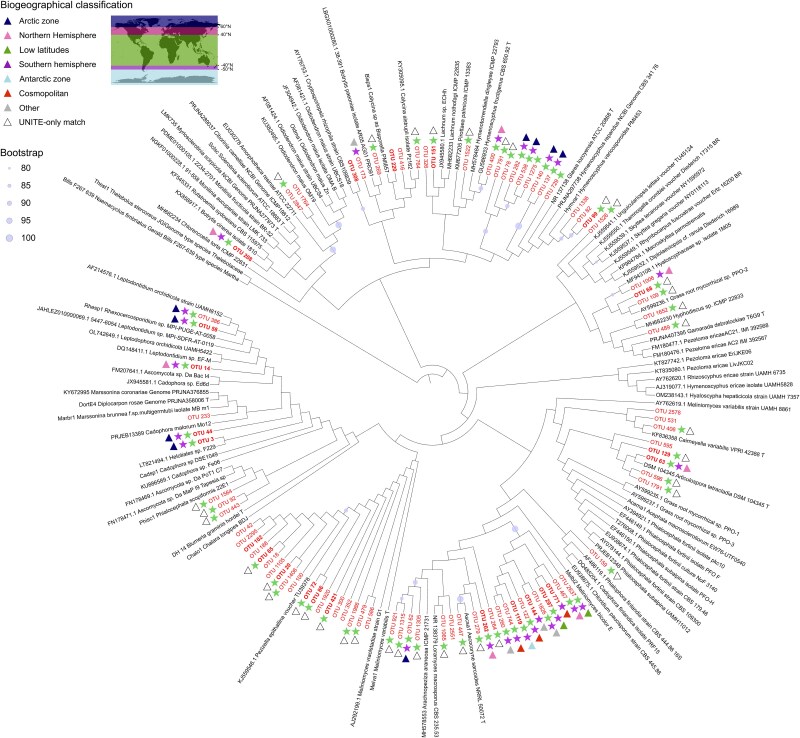
Phylogenetic tree of Helotiales ITS2 sequences from our study (plant- and non-plant-associated taxa, in red) and those retrieved from Bruyant *et al.* [5], in black. OTUs in bold indicate those defined as plant-associated. Coloured stars indicate OTUs that have a match in UNITE (green) and GlobalFungi (purple) at a sequence identity of ≥97%. Coloured triangles indicate their biogeographical range when known in GlobalFungi (≥97% identity threshold).

We further addressed the possibility of local speciation and diversification within the Helotiales that encompassed both OTUs whose sequences were already in public databases and non-documented ones. All of their corresponding ITS2 sequences were aligned to the ITS2 sequences of taxa representative of the known phylogenetic diversity of this order [5]. Although most internal branches of the resulting phylogenetic tree were poorly supported (bootstrap values below 80%; Fig. 4), it showed that both known and unknown Kerguelen OTUs were distributed over the entire tree. While several clusters of sequences encompassed Kerguelen OTUs and no reference sequences, all of these clusters included both already known and novel OTUs, thus not providing strong support for Kerguelen-specific diversification events provided that our taxon sampling was representative of the true Helotiales diversity in this archipelago.

### Known Kerguelen OTUs are more abundant in the world’s cold, carbon-rich and high-latitude terrestrial environments

Variance partitioning was conducted to assess the relative importance of geography versus environmental factors (annual mean temperatures and precipitations, SOC and soil pH) in shaping the global distribution of the known Kerguelen OTUs already recorded elsewhere on earth (287 non-plant-associated and 51 plant-associated taxa (Supplementary Fig. S4). Sample geographic coordinates explained 26% of the variation in OTU abundance, whereas environmental variables accounted for only 3% altogether with an additional 7% jointly explained by both environmental and spatial factors (*P* < .001). Among environmental predictors, MAT was the strongest predictor (*R*^2^ = 0.0500, *F* = 180.7, *P* = .001), followed by mean annual precipitations (*R*^2^ = 0.0240, *F* = 89.1, *P* = .001), soil pH (*R*^2^ = 0.0177, *F* = 66.8, *P* = .001), and SOC (*R*^2^ = 0.0086, *F* = 32.8, *P* = .001).

**Figure 5:**
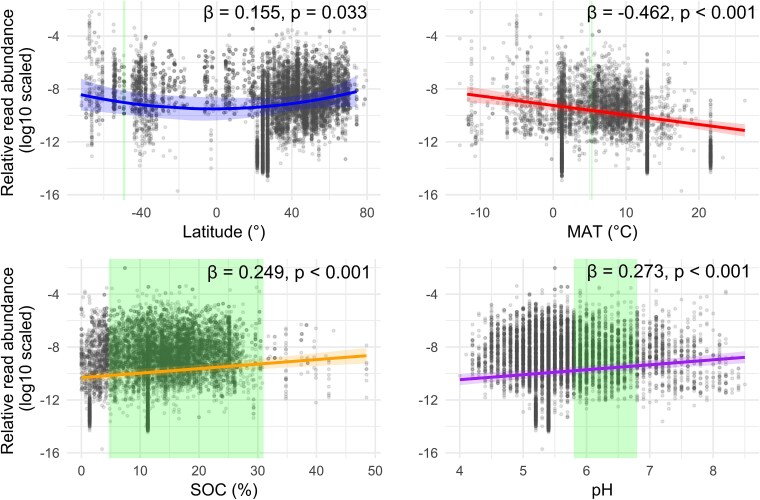
Latitudinal and environmental drivers of Kerguelen fungal OTUs abundance worldwide. Grey points represent log10-transformed OTU abundance in the different sites where they have been recorded globally; as extracted from the GlobalFungi database. Solid lines indicate the marginal effects of the variable studied (latitude, MAT, SOC, and soil pH) on fungal abundance as predicted by a linear mixed-effect model, with study identity, primers, and sample type as random effects. β coefficient (fixed-effect estimates) and *P*-values are reported for each significant variable. Shaded areas indicate the 95% confidence interval around the model prediction. Green shaded areas indicate the range of environmental values observed in the Kerguelen Islands. Details about the model output are reported in table S2.

A mixed-effects linear model enabled us to establish the direction of the relationships between global OTU abundance and each of the environmental variables (Fig. 5; Supplementary Table S2). We found significant associations between OTUs abundance in the GlobalFungi dataset and low MATs, high soil carbon content, higher pH values and higher latitudes.

### Latitudinal and biogeographic distribution of the already known plant- and non-plant-associated taxa

Of the OTUs identified in GlobalFungi, and therefore for which we can infer a known geographic distribution, a larger proportion (13%) of the non-plant-associated taxa had a cosmopolitan distribution, while the plant-associated ones had, seemingly, more restricted distribution ranges (less than 4% classified as cosmopolitans) (Fig. 3). Example of different distribution patterns are illustrated in Supplementary Fig. S6 for four different OTUs observed in the Kerguelen. Regarding plant-associated taxa, we observed differences among host plant species. While the non-native *P. annua* was not significantly associated with more cosmopolitan taxa, it was significantly associated with a higher abundance of Northern Hemisphere OTUs than both *Poa kerguelensis* and *Pringlea antiscorbutica* (Kruskal–Wallis, χ^2^ = 10.30, df = 2, *P* = .0058) (Supplementary Fig. S5).

Further mapping OTUs’ distribution on the plant biomes delineated in Loidi *et al.* [56], revealed that several of the Kerguelen’s OTUs appeared to have a truly ubiquitous distribution, being present in 10 or 11 biomes (including the NA category, corresponding to areas without vegetation cover such as Antarctica and Greenland). Others had however a much more restricted distribution being in several cases seemingly restricted to the tundra and Antarctic biomes (Supplementary Table S3;  Supplementary  Fig.  S6).

### Endemicity analysis

**Figure 6:**
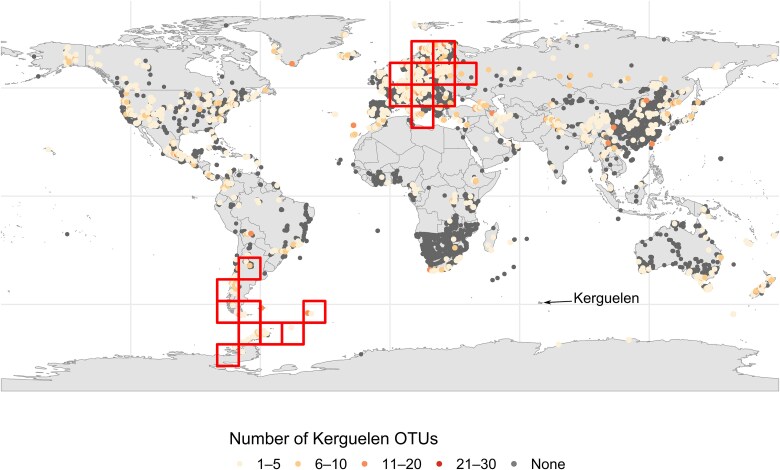
Geographic distribution of studies from the GlobalFungi database (as of January 2025). Dark grey dots represent all studies in the database that amplified the full ITS or ITS2 region of the ITS rDNA gene, while coloured dots (from light yellow to red) indicate studies that share at least one and up to 30 OTUs also identified in the Kerguelen Archipelago. Red squares represent the consensus endemicity zones, as identified using the VNDM approach.

In order to define potential endemicity areas from which the fungal taxa of Kerguelen could originate from, we conducted spatial VNDM analyses, based on the distribution of already known Kerguelen OTUs across the GlobalFungi dataset. As a result, two main consensus areas of fungal endemism emerged (Fig. 6 for the 10° × 10° grid). The first zone included regions of southern South America and the Antarctic Peninsula, while the second encompassed parts of Europe, particularly northern and central areas. These zones were consistently identified regardless of the spatial grid resolution used (see example in Supplementary Fig. S7 for the 5° × 5° grid). The number of taxa contributing to the definition of the southern hemisphere endemicity zone was almost twice the number of taxa that defined the European zone. Among the southern hemisphere taxa was *Mrakia frigida*, a psychrophilic basidiomycetous yeast isolated in Antarctica [58] (Tab. 1).

**Table 1:** List of OTUs giving the endemicity score to the two identified consensus area of endemism.

	**Phylum**	**Class**	**Order**	**Family**	**Species**	**VNDM endemicity score (0–1)**
**European endemicity zone**						
OTU_1437	Glomeromycota	Glomeromycetes	Diversisporales	Acaulosporaceae	*Acaulospora brasiliensis*	0.667
OTU_1782	Ascomycota	NA	NA	NA	NA	1
OTU_324	Mortierellomycota	NA	NA	NA	NA	0.667
OTU_857	Basidiomycota	Tremellomycetes	Tremellales	Tremellaceae	NA	0.667
OTU_1481	Ascomycota	Sordariomycetes	Atractosporales	Atractosporaceae	*Atractospora sp.*	0.750
OTU_1573	Chytridiomycota	Rhizophydiomycetes	Rhizophydiales	Halomycetaceae	NA	0.667
OTU_400	Ascomycota	Leotiomycetes	Helotiales	Helotiaceae	*Hymenoscyphus caudatus*	0.625
OUT_197	Basidiomycota	Ustilaginomycetes	Urocystidales	Urocystidaceae	*Urocystis sp.*	0.643
**Southern America/Antarctic Peninsula endemicity zone**						
OTU_1038	Ascomycota	NA	NA	NA	NA	1
OTU_1287	Mortierellomycota	Mortierellomycetes	Mortierellales	Mortierellaceae	*Mortierella turficola*	0.750
OTU_2186	Basidiomycota	Microbotryomycetes	Kriegeriales	Camptobasidiaceae	NA	0.8
OTU_558	Ascomycota	Sordariomycetes	NA	NA	NA	1
OTU_783	Ascomycota	NA	NA	NA	NA	1
OTU_1136	Mortierellomycota	Mortierellomycetes	Mortierellales	Mortierellaceae	*Linnemannia amoeboidea*	1
OTU_1837	Ascomycota	Lecanoromycetes	Lecanorales	Pilocarpaceae	*Micarea lapillicola*	0.833
OTU_536	Ascomycota	NA	NA	NA	NA	0.875
OTU_658	Basidiomycota	Pucciniomycetes	Platygloeales	Eocronartiaceae	*Eocronartium sp.*	1
OTU_917	Ascomycota	NA	NA	NA	NA	0.833
OTU_1337	Basidiomycota	Agaricomycetes	NA	NA	NA	0.750
OTU_280	Ascomycota	Leotiomycetes	NA	NA	NA	1
OTU_2202	Basidiomycota	Microbotryomycetes	Kriegeriales	Camptobasidiaceae	NA	0.750
OTU_46	Basidiomycota	Tremellomycetes	Cystofilobasidiales	Mrakiaceae	*Mrakia frigida*	1
OTU_682	Glomeromycota	Glomeromycetes	Diversisporales	Acaulosporaceae	*Acaulospora nivalis*	1

The endemicity score corresponds to the value given by the VNDM algorithm. It ranges from 0, meaning not found in the area to 1, meaning exclusive to the area. Values for the analysis performed with a grid mesh of 10° × 10° latitude × longitude.

## Discussion

### Low apparent level of fungal endemism in the Kerguelen

While the global number of fungal species remains a matter of debate [59–61], our molecular study indicates that a large majority (at least 60%) of fungal OTUs detected in Kerguelen soils and plant roots have previously been recorded in other regions. This data needs however to be interpreted cautiously in terms of its contribution to the debate of the real magnitude of fungal diversity. We have indeed used a single barcode sequence (ITS2), amplified with a single primer pair and used an arbitrary sequence identity threshold of 97% to delineate molecular taxa. Each of these three approaches is likely to bias estimate the true species richness in the ecosystem [62]. Furthermore, Kerguelen’s cold climate and the nature of its herbaceous, tundra-like vegetation make it similar to biogeographical areas naturally characterized by low fungal diversity [14].

In the case of the Kerguelen, we further tested the impact of grouping unique sequences (ASVs) to define OTUs on molecular taxon discovery rate (see Supplementary Fig. S2 and S3). We observed that an overwhelming majority of Kerguelen ASVs (95%), affiliated to known OTUs, had also already been recorded elsewhere on the globe, indicating that they are unlikely to represent technical artefacts, but also that our conclusions based on the occurrence of OTUs are robust. This observation also suggests repeated and independent colonization events of the Kerguelen by the different OTUs, including OTUs affiliated for example to the Glomeromycota that produce very large spores, which are arguably unlikely to be wind-dispersed to remote locations [11, 18].

Sampling protocols based on the collection of small quantities of soil (a few grams or less) are also more likely to highlight the diversity of abundant and uniformly distributed taxa and to underestimate the number of less frequent ones with a patchy spatial distribution [63]. This latter argument is supported by the observation that the percentage of ‘known OTUs’ (with sequences already present in the GlobalFungi and/or UNITE databases) is lowest among low abundance OTUs (third and fourth quartiles of the rank abundance distribution).

Despite all these limitations, which must be taken into account, our data suggest that the global efforts to characterize soil microbial diversity into structured public databases [50, 64, 65] could soon provide a comprehensive overview of the most abundant and globally distributed microbial taxa, including fungal ones, in soils. Such knowledge would enable targeted functional studies to assess their essential roles in soil biology, especially for large-scale studies.

As for OTUs seemingly specific to the Kerguelen, these may also be taxa with a wider distribution but not yet observed elsewhere, or OTUs that are truly endemic to these islands. The proportion of known versus unknown (<97% ITS2 sequence identity) varies greatly between databases used for comparisons and the taxonomical group considered. For example, for the UNITE database Eurotiales showed the highest mean sequence identity, whereas Lecanorales had the lowest (Supplementary Fig. S8). As a point of comparison, although our study recovered more than 60% of fungal OTUs with close matches in UNITE and/or GlobalFungi (≥97% sequence identity), 84% of the OTUs in Ercole *et al.* [66], who investigated soils from alpine (Italy, Europe) wetlands, corresponded to sequences already present in at least one of the two databases (Supplementary Fig. S9). This difference in percentages between our study and the one of Ercole *et al.* was significant for both inspected databases (Pearson’s χ^2^; UNITE, χ^2^ = 177.85, *P* < 2.2 × 10^−16^; GlobalFungi, χ^2^ = 98.52, *P* < 2.2 × 10^−16^). This suggests that geographical areas that have been little studied (in this case, the Kerguelen Islands) may still harbour a higher percentage of endemic taxa compared to areas that have already been well studied, such as Europe. In line with this pattern, recent studies highlighted the presence of fungi that are unique and specific to the Antarctic region, revealing a certain degree of endemism, even at a high taxonomic rank [67].We explored this possibility in the case of Helotiales, one of the most abundant and species-rich orders in the Kerguelen and a group frequently associated with plants as pathogens, mycorrhizal species or endophytes with documented beneficial functions [5, 68]. Their associations with two endemic plants could have led to speciation and diversification events in Kerguelen [69]. Our ITS2-based phylogenetic analysis did not reveal any such speciation events (Fig. 4). Although these analyses could be extended to all other taxonomic groups, the limited number of taxa revealed for each of them appears to be a limiting factor in detecting local diversification events. Given that ITS2 offers limited power to resolve deep phylogenetic structure, these results must be interpreted with caution. Future work could help refine the evolutionary relationships presented here by studying other markers with stronger phylogenetic signal (e.g. LSU, RPB2) or by applying phylogenetically informed placement tools such as q2-fragment-insertion [70–72].

### Affinities and putative origins of the Kerguelen’s mycoflora

Taken together, the above observations suggest that the fungal flora of the Kerguelen Islands is not as isolated as the extreme geographic isolation of these islands would suggest. We have carried out a series of additional analyses that enable us to propose possible historical routes of colonization of the Kerguelen Islands by fungal species observed elsewhere on earth.

Firstly, correlative analyses show that the fungal species present both in Kerguelen and elsewhere are naturally more abundant at high latitudes and associated with low temperatures, higher soil pH, and high levels of organic matter. These characteristics reflect relatively well the geographical location, edaphic and climatic conditions prevailing in Kerguelen.

With regard to the overall geographical distribution of the species, we identified soil cosmopolitan species present in all latitudes and in very contrasted plant biomes. However, these species are the minority among those associated with Kerguelen plants. Ubiquitous taxa, belonging to the Ascomycota, have already been reported in the literature [12]. Very few of them are present in our dataset (Supplementary Fig. S10). Beyond these cosmopolitan species, the majority of species are found at high latitudes (>40°N or >50°S), particularly in the Arctic and Antarctic regions. These data are corroborated by the observation of a large number of taxa associated with the tundra plant biome. Altogether, these observations apparently support the view of a global dispersal of fungal species around the globe, from pole to pole [73], and the selection of taxa adapted to the local pedoclimatic conditions (environmental filtering). This observation also applies to taxa associated with plants, including two endemic species. It therefore seems that the geographical isolation that certainly led to allopatric speciation of plant taxa did not result in the emergence of new fungal taxa, as suggested, for example, in plants and ectomycorrhizal fungi [69] or other taxa typical of the Antarctic bioregion [74].

However, geographically informed analyses temper these conclusions. Indeed, a spatial biogeographical analysis has succeeded in delineating two distinct geographical areas: one in Europe and the other in the Antarctic/sub-Antarctic region (Fig. 6), within which the fungal flora shows affinities with that of the Kerguelen Islands. These two zones are not equivalent insofar as the fungal taxa contributing to the delimitation of each are not the same. These same zones emerge regardless of the sampling grid chosen, suggesting that the European zone, characterized by a very high sampling point density, is not the result of a technical artefact. Furthermore, other geographical zones in the Northern Hemisphere (in North America or northern China) where the sampling point density is also high have not shown any significant affinities with the mycoflora of Kerguelen.

The biogeographical zone located between the sub-Antarctic peninsula and the southern tip of the South American continent suggests that the landmasses of the sub-Antarctic zone are connected to each other. To confirm this hypothesis, it would be necessary to obtain metabarcoding data from soils across all the islands scattered throughout this zone. Such a study was recently carried out [24], but was conducted using fungal ITS1 amplification, making it impossible to cross-reference the data obtained with those obtained in our study using the ITS2 barcode sequence.

With regard to the European biogeographical zone, it is interesting to note that maritime traffic between Kerguelen and the rest of the world has been mainly limited to Europe from the end of the 18th century, and more specifically Norway, the UK, and France. Historically, whaling and seal hunting bases were established in Kerguelen by Norwegians and the English, respectively [75]. Unsuccessful attempts at agriculture and livestock farming were made, and for the last 75 years human settlement has been limited to research and military activities, to the exception of fish farming trials in the 1980s and food cultivations in greenhouses for the base until the 1980s in Port-aux-Français. Despite limited exchanges confined to specific coastal areas, these activities have led to the importation, mainly from Europe, of construction materials, food, animals, fodder, and potting soil. It can be hypothesized that these exchanges would have permitted, as has been documented for plants and insects [19, 76], the accidental importation of numerous European fungal species adapted to local soil and climate conditions. These would have colonized the entire archipelago, including the fellfields where there are no human settlements.

## Conclusions

In conclusion, the current mycoflora of Kerguelen seems to be the result of a combination of several factors: the historical geographical isolation of the Antarctic/sub-Antarctic region, recent colonization (since the 18th century) through biological invasions mainly coming from Europe (anthropogenic component) and, finally, the global dispersal of a number of ubiquitous species with no specific geographical origin. For the latter two components, correlations with various environmental parameters demonstrate the role of environmental filtering in successful establishment in the Kerguelen fellfields.

Beyond this temporal biogeographical dynamic, the study of fungi in the soil and plant roots of Kerguelen reveals that around 80% of the dominant OTUs present at the sites studied have already been recorded elsewhere on the globe. Such a high value could suggest that the observation of abundant fungal taxa in soils could very quickly reach a plateau. This conclusion should be confirmed by additional studies of isolated islands at lower latitudes, where milder environmental conditions could allow for the development of greater fungal biodiversity.

Finally, analyses conducted at the sequence variant level temper this conclusion. Although Kerguelen-specific ASVs are observed in a minority of known OTUs, they sometimes dominate in terms of read abundance within these OTUs, suggesting local diversification or genetic isolation of taxa that are otherwise distributed globally. These results highlight the importance of conducting analyses at both the OTU and ASV levels to understand fungal endemism.

## Supplementary Material

FigureS1_ycag095

FigureS2_ycag095

FigureS3_ycag095

FigureS4_ycag095

FigureS5_ycag095

FigureS6_ycag095

FigureS7_ycag095

FigureS8_ycag095

FigureS9_ycag095

FigureS10_ycag095

clean_supplementary_ycag095

## Data Availability

*Poa kerguelensis* and *Pringlea antiscorbutica* data can be found on the European Nucleotide Archive (EMBL-ENA) under accession number PRJEB89188 and *P. annua* data can be found under the accession number PRJEB90454.
