# Supplementary figures and images for: So far but so close: the biogeography of soil and plant-associated fungi in one of the most remote landmasses on Earth
Source: ISME Commun. 2026 Apr 14;6(1):ycag095. doi: 10.1093/ismeco/ycag095 (PMC13155106; doi:10.1093/ismeco/ycag095)

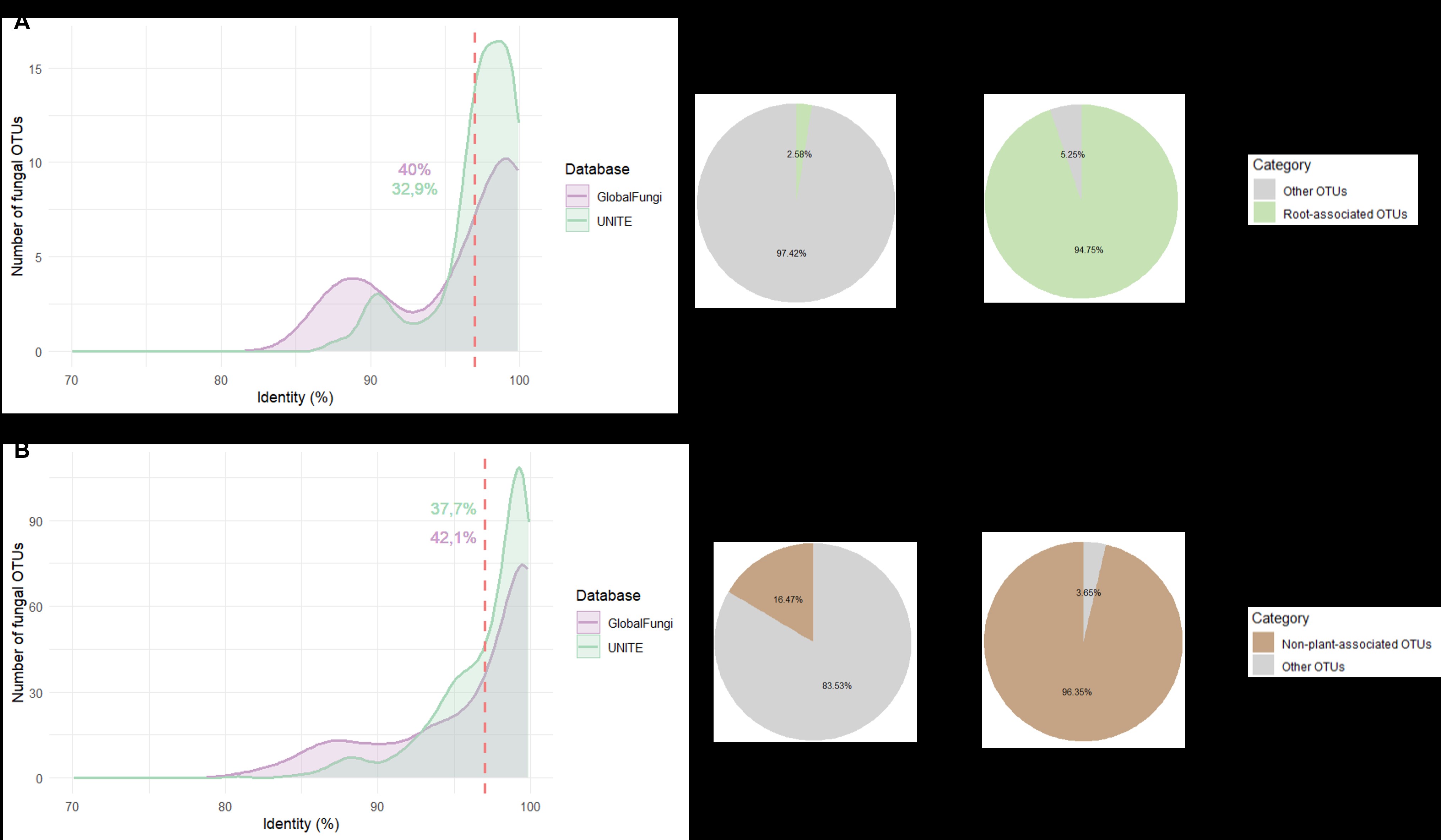

Supplement: FigureS1_ycag095 [file figures1_ycag095.jpeg]

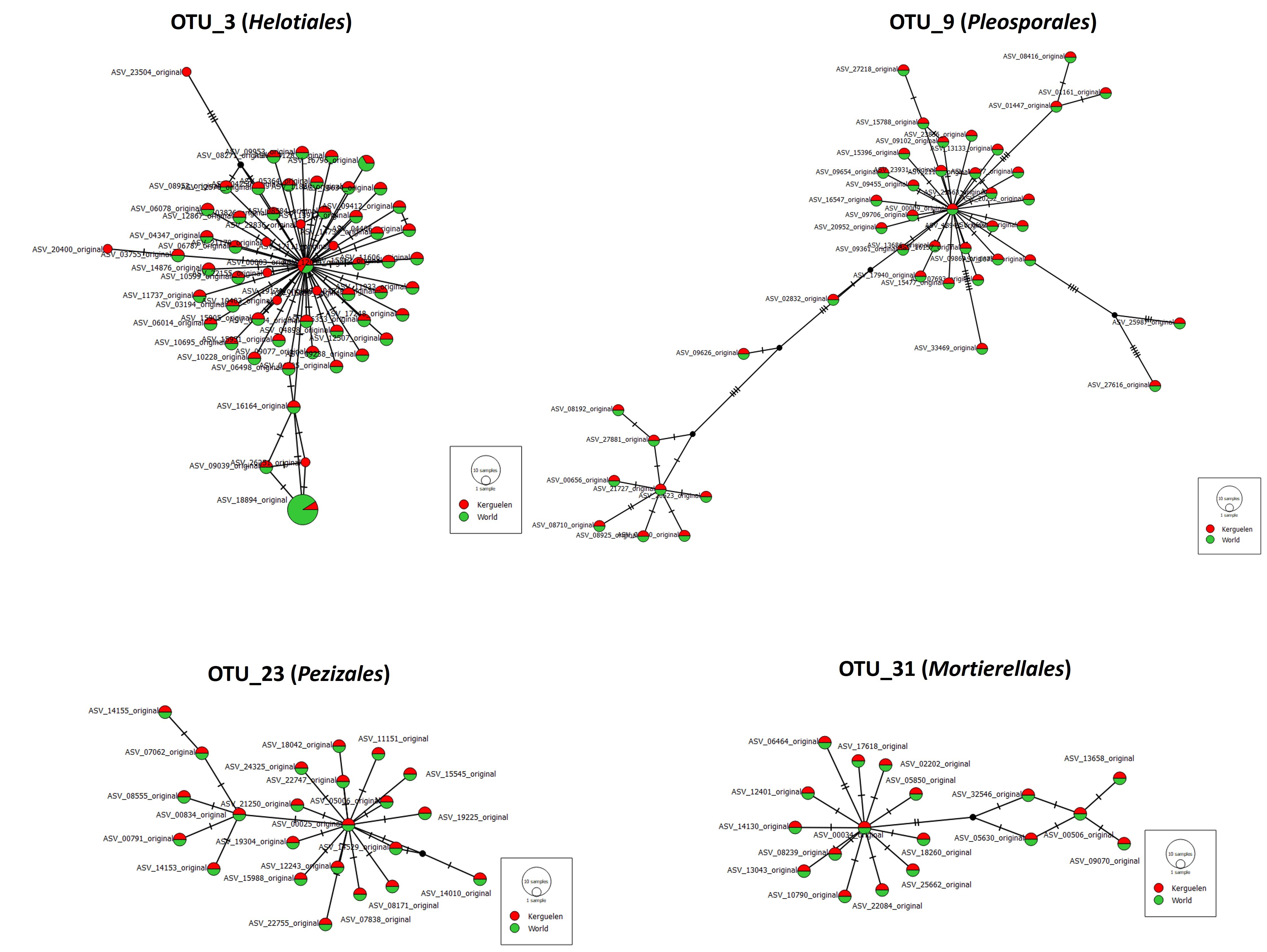

Supplement: FigureS2_ycag095 [file figures2_ycag095.jpeg]

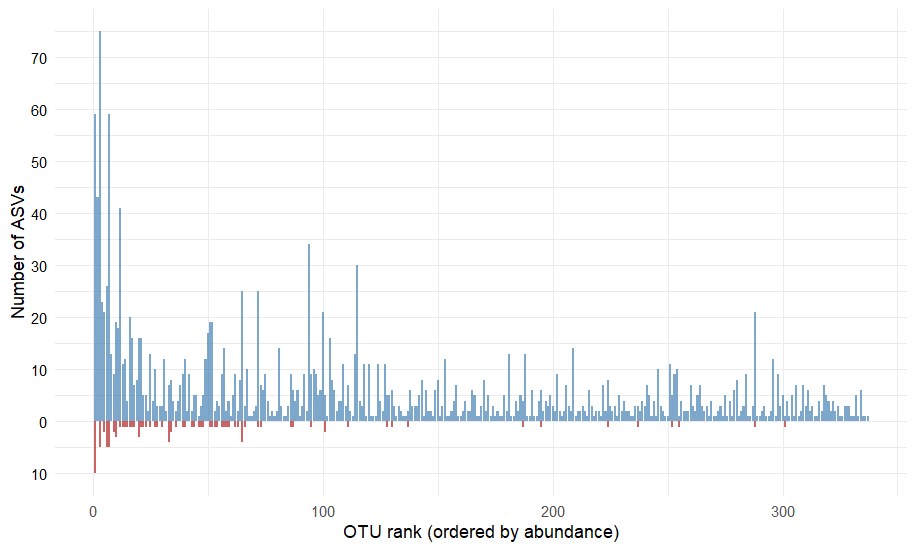

Supplement: FigureS3_ycag095 [file figures3_ycag095.jpeg]

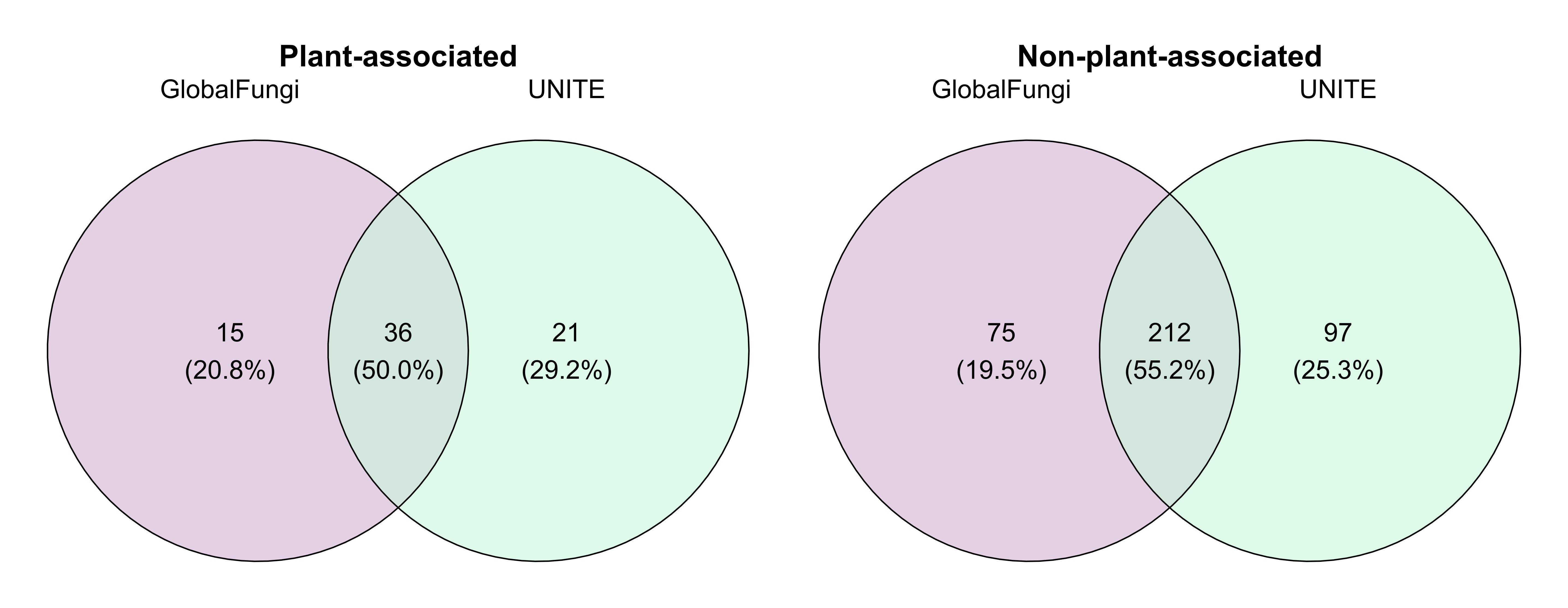

Supplement: FigureS4_ycag095 [file figures4_ycag095.jpeg]

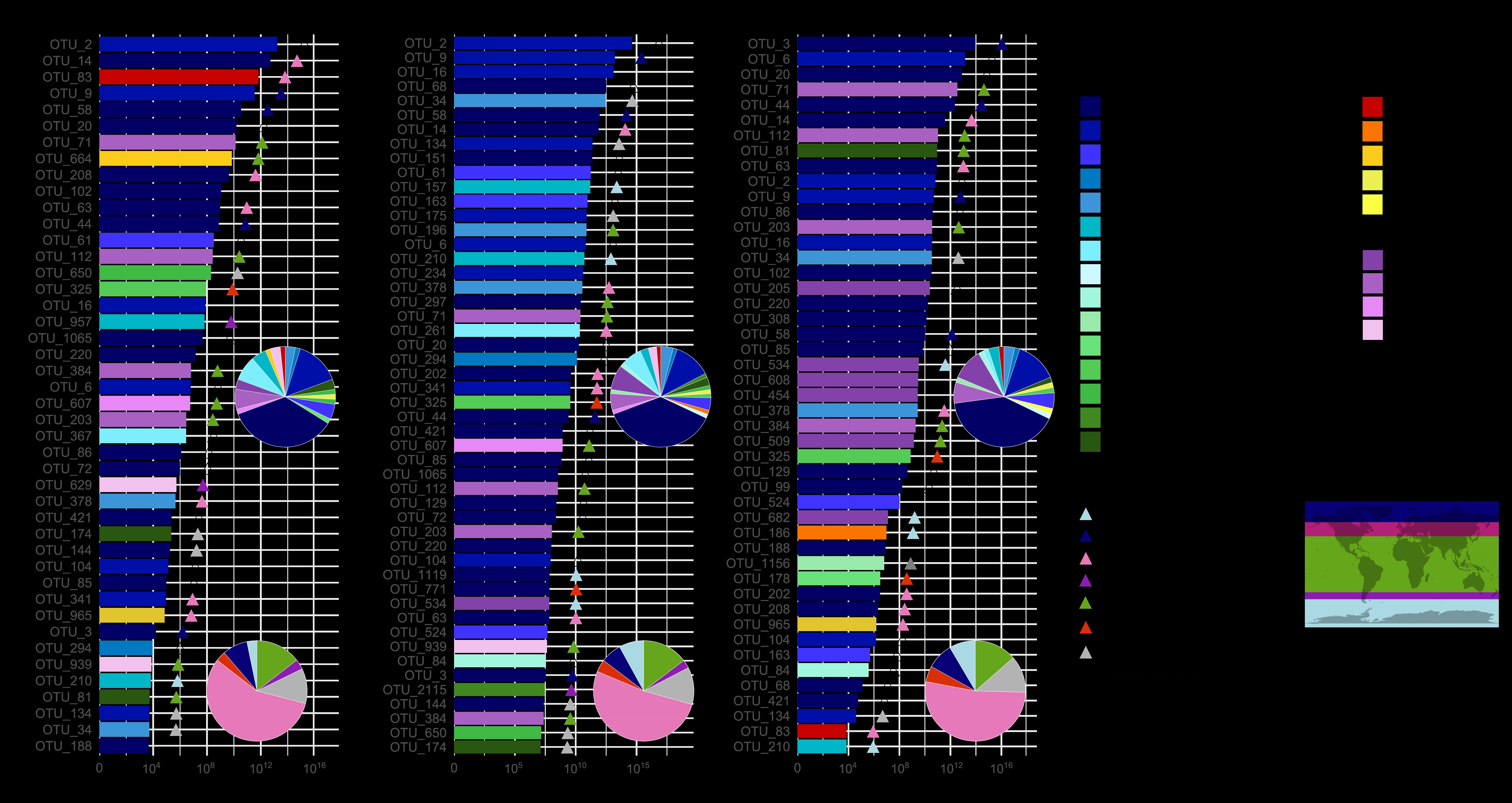

Supplement: FigureS5_ycag095 [file figures5_ycag095.jpeg]

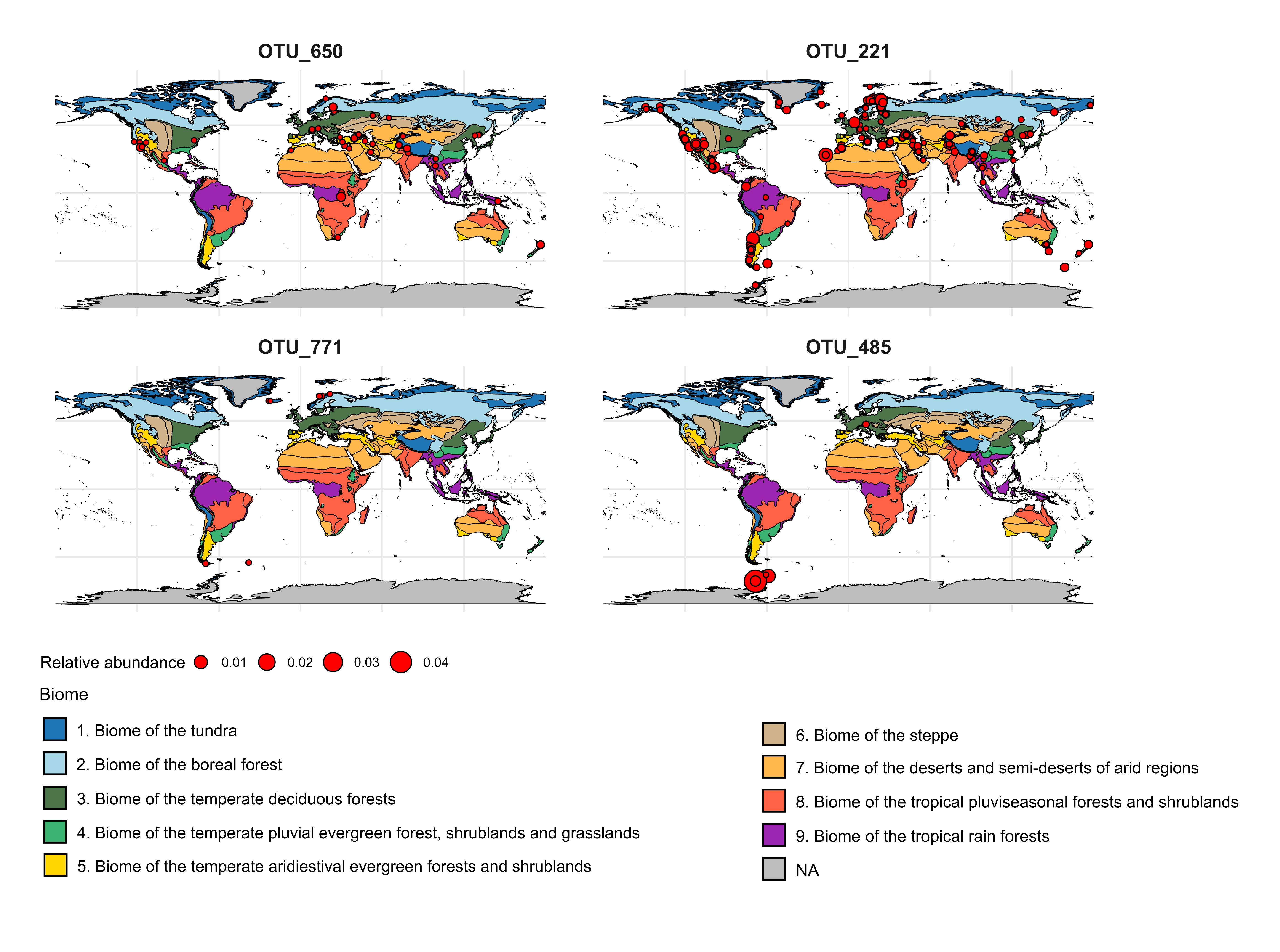

Supplement: FigureS6_ycag095 [file figures6_ycag095.jpeg]

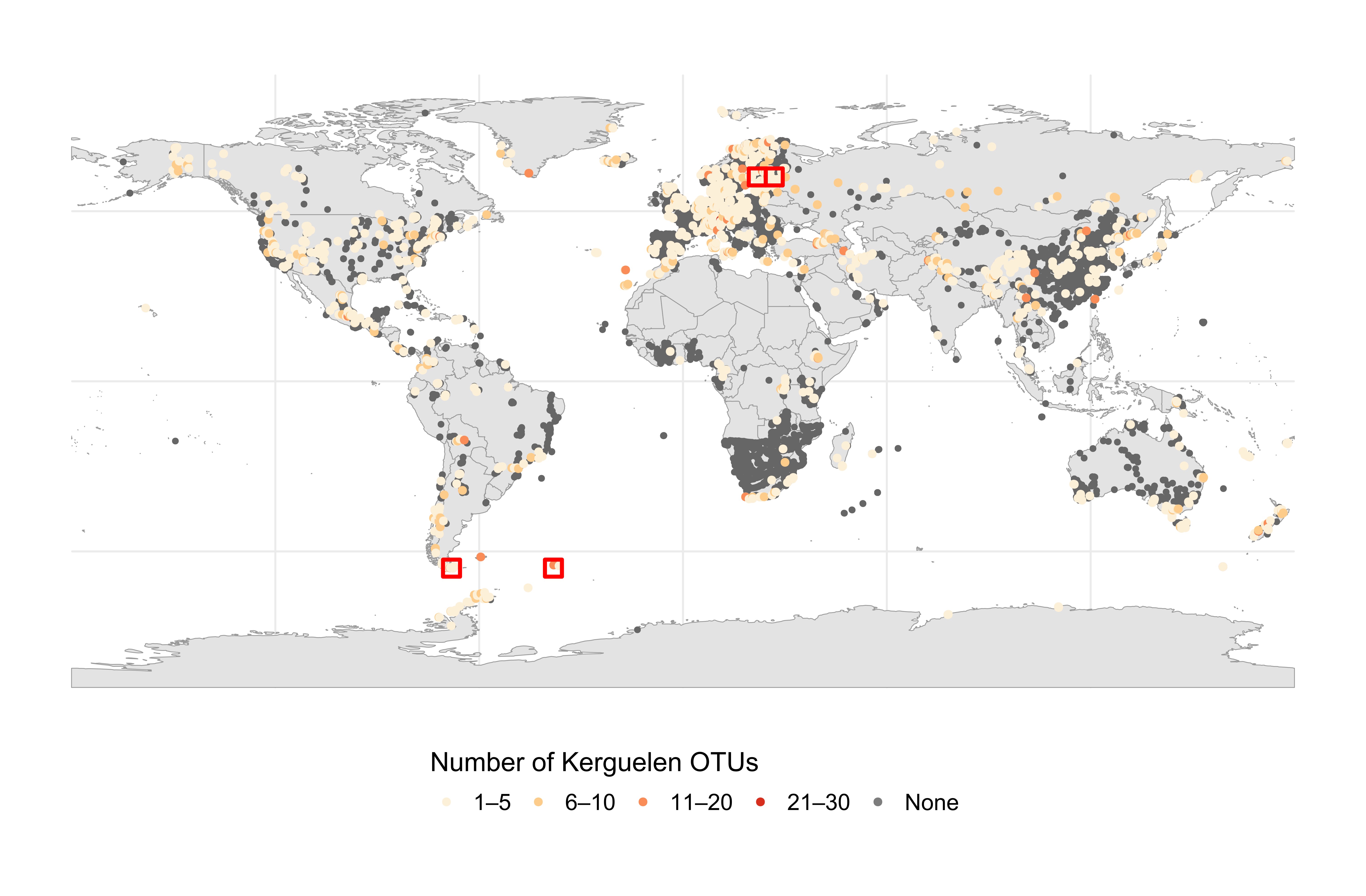

Supplement: FigureS7_ycag095 [file figures7_ycag095.jpeg]

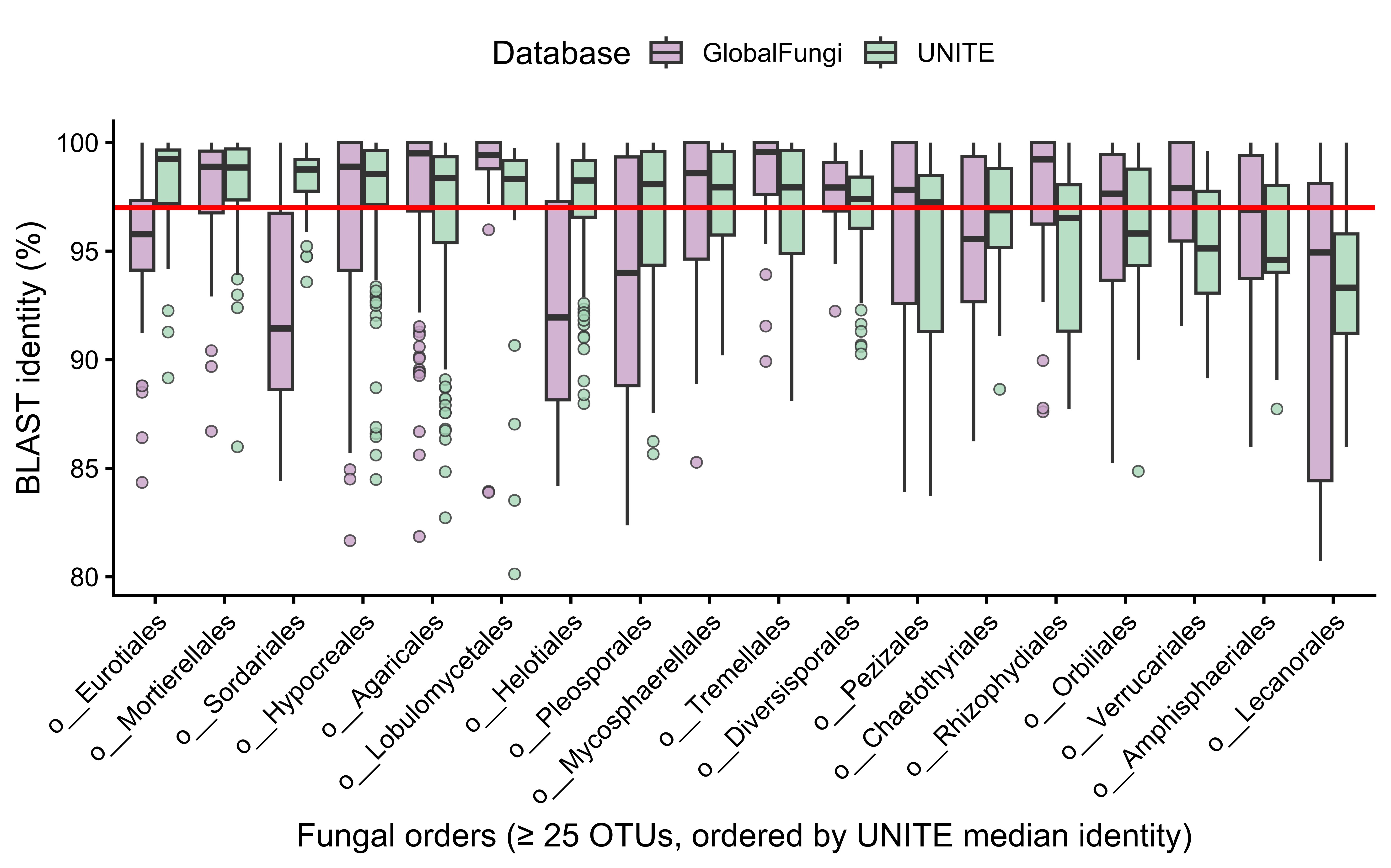

Supplement: FigureS8_ycag095 [file figures8_ycag095.jpeg]

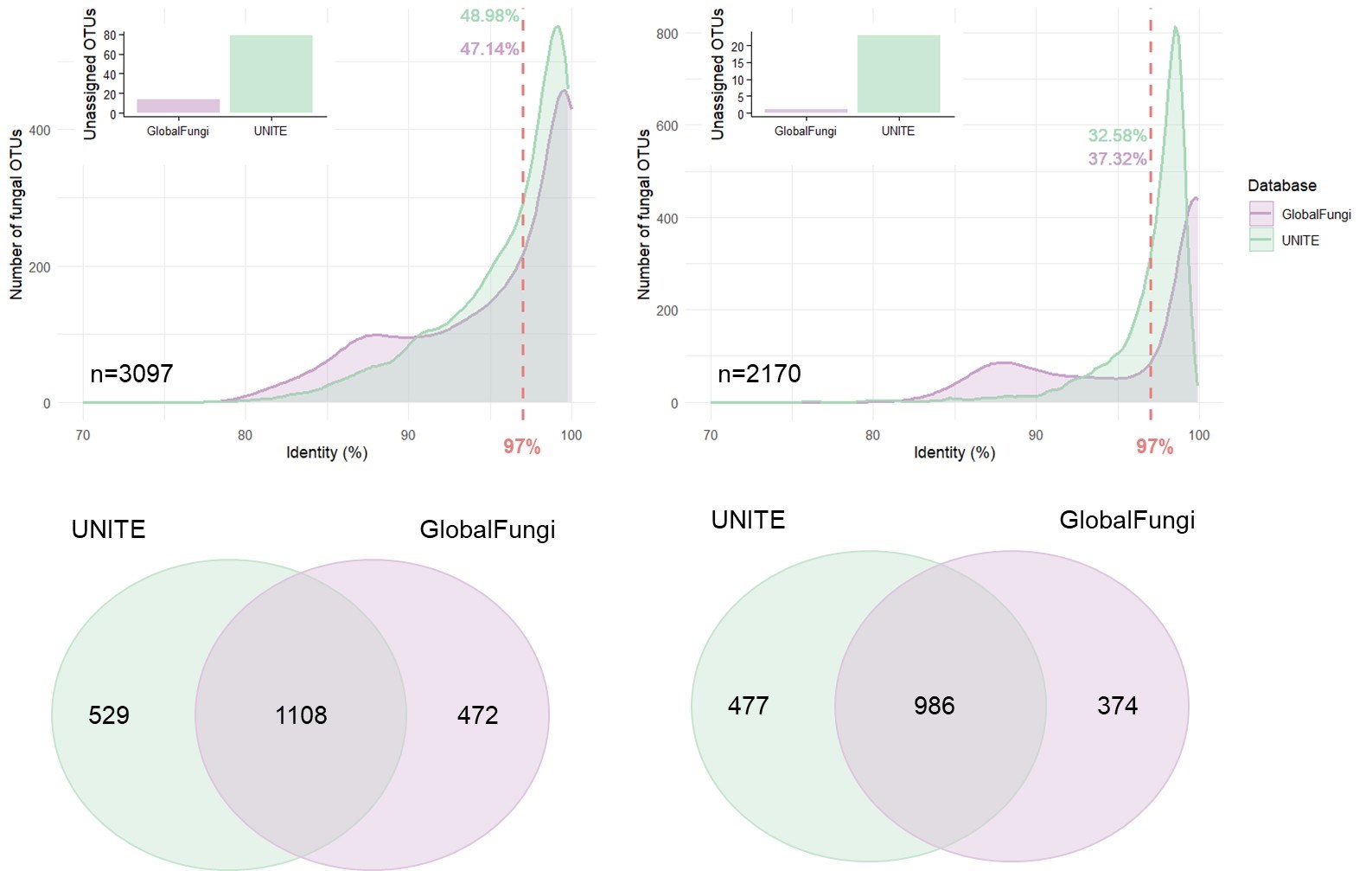

Supplement: FigureS9_ycag095 [file figures9_ycag095.jpeg]

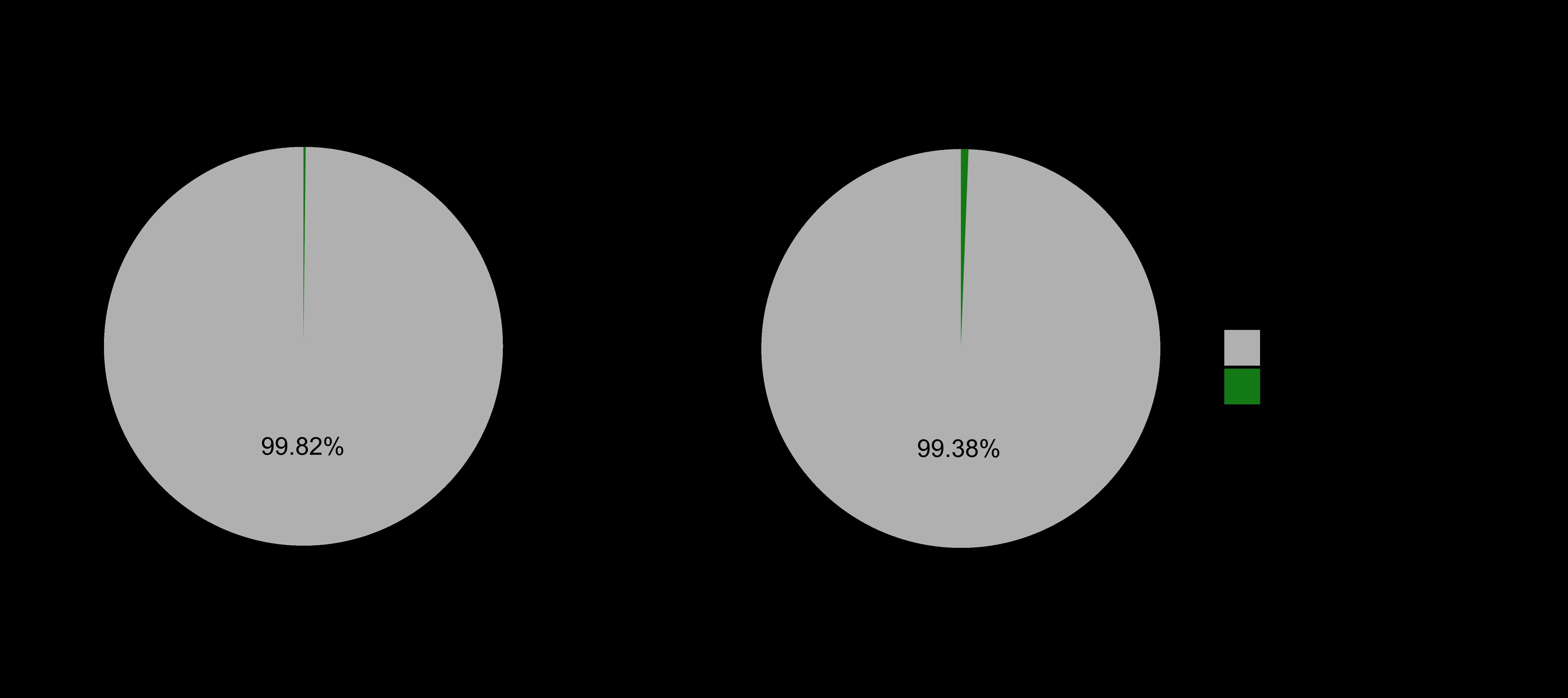

Supplement: FigureS10_ycag095 [file figures10_ycag095.jpeg]
