# Supplementary material for: So far but so close: the biogeography of soil and plant-associated fungi in one of the most remote landmasses on Earth
Source: ISME Commun. 2026 Apr 14;6(1):ycag095. doi: 10.1093/ismeco/ycag095 (PMC13155106; doi:10.1093/ismeco/ycag095)
Supplement: clean_supplementary_ycag095 [file clean_supplementary_ycag095.docx]

**SUPPLEMENTARY MATERIALS**

So far but so close: the biogeography of soil and plant-associated fungi in one of the most remote landmasses on Earth

**AUTHORS:** Constance Bertrand, Françoise Binet, Martino Adamo, Marie-Claire Martin, Roland Marmeisse

Figure S1: Selected plant-associated (A) and non-plant-associated taxa (B), their proportion in terms of OTU richness and relative read abundance in our dataset and their occurrence in the global databases UNITE and GlobalFungi.

Figure S2: Haplotype networks of the top two plant-associated OTUs (OTU 3 and OTU 9) and non-plant-associated OTUs (OTU 23 and OTU 31) based on global sequence alignments. Each circle represents a unique haplotype (ASV), with circle size proportional to the number of sequences. Colours indicate geographic origin, and hatch marks represent mutational steps.

Figure S3: Total (blue) and Kerguelen-exclusive (red) ASVs per fungal OTU. Ranked fungal OTUs are shown with total ASV abundance (blue bars) and the number of ASVs exclusive to Kerguelen (corresponding to sequence without match at 100% identity (red bars)). OTUs are ranked by their total read abundance across all samples.

Figure S4: Shared and unique OTUs with ≥97% identity in GlobalFungi and UNITE databases. Unknown taxa (i.e., taxa blasted against a reference sequence with less than 97% identity) represent 16% of plant-associated and 23% of non-plant-associated OTUs.

Figure S5: Rank-abundance diagram showing the taxonomy and geographical classification of the 50 most abundant root-associated fungal taxa for each of the three studied plant species. Coloured triangles indicate a ≥97% identity match against the GlobalFungi database, with an associated biogeographical origin.
Void triangles indicate a ≥97% identity match only in the UNITE database (and <97% in GlobalFungi), meaning no geographical metadata is available. Taxa with no triangle had <97% identity matches in both databases. Biogeographical classes were defined purely on the latitudinal distribution of matched taxa of the GlobalFungi database. For each OTU, we calculated the minimum and maximum sampling latitude and assigned it to one of the following classes: cosmopolitan (latitudinal range of over 60° and spanning both hemispheres), Arctic zone (≥60° N), Antarctic zone (≤-50°N), Northern Hemisphere (between 40° and 60°N), Southern Hemisphere (between -40° and -50°N), or low latitudes (between -40° and 40°N). Taxa not meeting these criteria were classified as Other. Pie charts represents the proportion of each order (top) and each biogeographical class (bottom) across the whole dataset.

Figure S6: Spatial occurrence of two cosmopolitan OTUs (OTU_650 and OTU_221, top panels) and two polar-restricted OTUs (OTU_771 and OTU_485, bottom panels). Points represent sampling locations (data from GloablFungi), with size scaled to relative abundance. Biomes are coloured according to the biome delineation by Loidi et al [1].

Figure S7: Geographic distribution of studies from the GlobalFungi database. Dots represent all studies in the database that amplified the full ITS or ITS2 region of the ITS rDNA gene. Coloured dots (from light yellow to red) indicate studies that share at least one and up to 30 OTUs also identified in the Kerguelen Archipelago. Red squares represent the consensus endemicity zones, as identified using the VNDM approach. For this spatial analysis, the 5 x 5° mesh grid was used.

Figure S8: UNITE and GlobalFungi differ with respect to their coverage of several major fungal orders, as illustrated by the distribution of percentages of sequence identity between Kerguelen OTU sequences and their best matches in the two databases. Only orders with more than 25 OTUs were considered. Orders are presented on the x-axis by descending order of median identity blasted against UNITE. The red horizontal line corresponds to the 97% identity threshold used in this study.

Figure S9: Overview of “known” and “unknown” taxa (red dotted line corresponding to the identity threshold of 97%) proportions for our study (left) and Ercole et al. [2] (right) across UNITE and GlobalFungi databases and known taxa assignment overlap visualized with Venn diagrams. Percentages next to the 97% identity threshold line correspond to the proportions of Kerguelen OTU sequences displaying a % identity below 97% against any sequence present in either the UNITE or GlobalFungi database.

Figure S10: Relative read abundance of ubiquitous Ascomycota, as delineated in [3], and the proportion of OTUs they represent in our dataset.

Table S1: Contribution of Kerguelen-specific ASVs to fungal OTUs. For each fungal OTU, the table reports its taxonomy at the order level, the total number of ASVs comprised in the OTU, the number of ASVs that are Kerguelen-specific (i.e., which sequences differ from their closest GlobalFungi match) and the cumulative relative read abundance of these Kerguelen-specific ASVs within the OTU. The remaining columns provide for each Kerguelen-specific ASV its relative read abundance within the OTU.

| **OTU** | **Taxonomy (order level)** | **Total no. of ASVs** | **No. of Kerguelen-specific ASVs** | **Cumulative read abundance of Kerguelen-specific ASVs** | **1** | **2** | **3** | **4** | **5** | **6** | **7** | **8** | **9** | **10** |
| --- | --- | --- | --- | --- | --- | --- | --- | --- | --- | --- | --- | --- | --- | --- |
| OTU_3 | Helotiales | 59 | 10 | 0.0173 | ASV_17111 0.0030 | ASV_19174 0.0024 | ASV_19403 0.0023 | ASV_20400 0.0020 | ASV_21178 0.0018 | ASV_22155 0.0016 | ASV_22836 0.0015 | ASV_23504 0.0014 | ASV_26251 0.0010 | ASV_34573 0.0003 |
| OTU_14 | Helotiales | 75 | 5 | 90.4323 | ASV_00014 36.6168 | ASV_00016 35.0276 | ASV_00059 13.4096 | ASV_00276 2.8289 | ASV_00297 2.5495 |  |  |  |  |  |
| OTU_34 | Sordariomycetes ord Incertae sedis | 26 | 5 | 99.1743 | ASV_00037 78.1209 | ASV_00142 20.9653 | ASV_09893 0.0371 | ASV_11553 0.0268 | ASV_12151 0.0242 |  |  |  |  |  |
| OTU_58 | Helotiales | 59 | 5 | 43.6132 | ASV_00069 32.0267 | ASV_00346 5.8209 | ASV_00414 4.5933 | ASV_01422 0.8508 | ASV_02601 0.3216 |  |  |  |  |  |
| OTU_155 | NA | 25 | 4 | 0.1516 | ASV_16001 0.0452 | ASV_16896 0.0401 | ASV_18127 0.0343 | ASV_18604 0.0321 |  |  |  |  |  |  |
| OTU_175 | Pleosporales | 7 | 4 | 0.5326 | ASV_09790 0.2822 | ASV_12660 0.1642 | ASV_21976 0.0462 | ASV_23175 0.0399 |  |  |  |  |  |  |
| OTU_63 | Helotiales | 19 | 3 | 81.2292 | ASV_00078 38.1873 | ASV_00097 31.3726 | ASV_00262 11.6693 |  |  |  |  |  |  |  |
| OTU_77 | Pleosporales | 16 | 3 | 62.4025 | ASV_00099 48.9347 | ASV_00331 13.2746 | ASV_05368 0.1932 |  |  |  |  |  |  |  |
| OTU_241 | Verrucariales | 5 | 2 | 74.8465 | ASV_00315 74.8079 | ASV_24422 0.0386 |  |  |  |  |  |  |  |  |
| OTU_71 | Entrophosporales | 21 | 2 | 33.6504 | ASV_00124 24.0618 | ASV_00298 9.5886 |  |  |  |  |  |  |  |  |
| OTU_90 | NA | 9 | 2 | 61.9149 | ASV_00114 44.6774 | ASV_00293 17.2375 |  |  |  |  |  |  |  |  |
| OTU_98 | Leucosporidiales | 8 | 2 | 97.4614 | ASV_00128 97.3506 | ASV_11787 0.1107 |  |  |  |  |  |  |  |  |
| OTU_112 | Entrophosporales | 3 | 1 | 99.9317 | ASV_00147 99.9317 |  |  |  |  |  |  |  |  |  |
| OTU_117 | Hypocreales | 20 | 1 | 42.5534 | ASV_00153 42.5534 |  |  |  |  |  |  |  |  |  |
| OTU_120 | Pleosporales | 3 | 1 | 99.5696 | ASV_00156 99.5696 |  |  |  |  |  |  |  |  |  |
| OTU_140 | Helotiales | 16 | 1 | 23.1405 | ASV_00288 23.1405 |  |  |  |  |  |  |  |  |  |
| OTU_178 | Hypocreales | 14 | 1 | 97.4230 | ASV_00235 97.4230 |  |  |  |  |  |  |  |  |  |
| OTU_1831 | Archeorhizomycetales | 1 | 1 | 100 | ASV_02805 100 |  |  |  |  |  |  |  |  |  |
| OTU_1835 | Microbotrymycetes ord Incertae sedis | 1 | 1 | 100 | ASV_02813 100 |  |  |  |  |  |  |  |  |  |
| OTU_184 | Pezizales | 9 | 1 | 63.6812 | ASV_00241 63.6812 |  |  |  |  |  |  |  |  |  |
| OTU_186 | Kriegeriales | 3 | 1 | 98.0121 | ASV_00243 98.0121 |  |  |  |  |  |  |  |  |  |
| OTU_1886 | Rozellomycota ord Incertae sedis | 5 | 1 | 1.6927 | ASV_15704 1.6927 |  |  |  |  |  |  |  |  |  |
| OTU_191 | Pezizales | 5 | 1 | 96.9186 | ASV_00248 96.9186 |  |  |  |  |  |  |  |  |  |
| OTU_196 | Sordariomycetes ord Incertae sedis | 2 | 1 | 99.8159 | ASV_00258 99.8159 |  |  |  |  |  |  |  |  |  |
| OTU_197 | Urocystidales | 2 | 1 | 99.7952 | ASV_00259 99.7952 |  |  |  |  |  |  |  |  |  |
| OTU_202 | Helotiales | 5 | 1 | 99.3187 | ASV_00266 99.3187 |  |  |  |  |  |  |  |  |  |
| OTU_203 | Entrophosporales | 1 | 1 | 100 | ASV_00267 100 |  |  |  |  |  |  |  |  |  |
| OTU_208 | Helotiales | 9 | 1 | 68.7587 | ASV_00274 68.7587 |  |  |  |  |  |  |  |  |  |
| OTU_210 | Atractosporales | 2 | 1 | 99.9361 | ASV_00277 99.9361 |  |  |  |  |  |  |  |  |  |
| OTU_215 | Hypocreales | 9 | 1 | 99.3498 | ASV_00283 99.3498 |  |  |  |  |  |  |  |  |  |
| OTU_221 | Holtermanniales | 9 | 1 | 54.0906 | ASV_00292 54.0906 |  |  |  |  |  |  |  |  |  |
| OTU_224 | Agaricales | 6 | 1 | 93.4934 | ASV_00296 93.4934 |  |  |  |  |  |  |  |  |  |
| OTU_227 | Myrmecridiales | 2 | 1 | 99.9551 | ASV_00301 99.9551 |  |  |  |  |  |  |  |  |  |
| OTU_230 | Rhizophydiales | 7 | 1 | 81.2779 | ASV_00304 81.2779 |  |  |  |  |  |  |  |  |  |
| OTU_235 | Agaricales | 19 | 1 | 38.4729 | ASV_00309 38.4729 |  |  |  |  |  |  |  |  |  |
| OTU_236 | Xylariales | 4 | 1 | 88.1124 | ASV_00310 88.1124 |  |  |  |  |  |  |  |  |  |
| OTU_237 | Geoglossales | 7 | 1 | 96.9117 | ASV_00311 96.9117 |  |  |  |  |  |  |  |  |  |
| OTU_239 | Verrucariales | 9 | 1 | 43.6369 | ASV_00313 43.6369 |  |  |  |  |  |  |  |  |  |
| OTU_240 | Mortierellales | 3 | 1 | 87.0212 | ASV_00314 87.0212 |  |  |  |  |  |  |  |  |  |
| OTU_246 | Hypocreales | 2 | 1 | 99.8423 | ASV_00320 99.8423 |  |  |  |  |  |  |  |  |  |
| OTU_254 | Urocystidales | 2 | 1 | 99.7677 | ASV_00329 99.7677 |  |  |  |  |  |  |  |  |  |
| OTU_255 | Verrucariales | 8 | 1 | 95.5755 | ASV_00330 95.5755 |  |  |  |  |  |  |  |  |  |
| OTU_260 | Helotiales | 12 | 1 | 57.7644 | ASV_00336 57.7644 |  |  |  |  |  |  |  |  |  |
| OTU_261 | Chaetothyriales | 4 | 1 | 98.4209 | ASV_00337 98.4209 |  |  |  |  |  |  |  |  |  |
| OTU_265 | Verrucariales | 19 | 1 | 48.3901 | ASV_00342 48.3901 |  |  |  |  |  |  |  |  |  |
| OTU_268 | Hypocreales | 5 | 1 | 98.0180 | ASV_00345 98.0180 |  |  |  |  |  |  |  |  |  |
| OTU_351 | Orbiliales | 6 | 1 | 0.0916 | ASV_18332 0.0916 |  |  |  |  |  |  |  |  |  |
| OTU_3630 | Rozellomycota ord Incertae sedis | 21 | 1 | 0.8012 | ASV_26582 0.8012 |  |  |  |  |  |  |  |  |  |
| OTU_392 | Cystofilobasidiales | 3 | 1 | 0.9958 | ASV_08674 0.9958 |  |  |  |  |  |  |  |  |  |
| OTU_45 | NA | 4 | 1 | 99.0524 | ASV_00050 99.0524 |  |  |  |  |  |  |  |  |  |
| OTU_46 | Cystofilobasidiales | 41 | 1 | 38.8447 | ASV_00051 38.8447 |  |  |  |  |  |  |  |  |  |
| OTU_47 | Mortierellales | 5 | 1 | 99.5221 | ASV_00052 99.5221 |  |  |  |  |  |  |  |  |  |
| OTU_48 | Cystofilobasidiales | 10 | 1 | 99.7551 | ASV_00053 99.7551 |  |  |  |  |  |  |  |  |  |
| OTU_50 | Hypocreales | 11 | 1 | 95.1377 | ASV_00056 95.1377 |  |  |  |  |  |  |  |  |  |
| OTU_62 | Helotiales | 5 | 1 | 93.4119 | ASV_00075 93.4119 |  |  |  |  |  |  |  |  |  |
| OTU_64 | Mortierellales | 12 | 1 | 92.5331 | ASV_00080 92.5331 |  |  |  |  |  |  |  |  |  |
| OTU_67 | Verrucariales | 25 | 1 | 82.0244 | ASV_00085 82.0244 |  |  |  |  |  |  |  |  |  |
| OTU_76 | Mortierellales | 7 | 1 | 99.5281 | ASV_00098 99.5281 |  |  |  |  |  |  |  |  |  |
| OTU_81 | Orbiliales | 13 | 1 | 99.0049 | ASV_00103 99.0049 |  |  |  |  |  |  |  |  |  |
| OTU_83 | Agaricales | 16 | 1 | 98.0883 | ASV_00105 98.0883 |  |  |  |  |  |  |  |  |  |
| OTU_96 | NA | 6 | 1 | 92.1048 | ASV_00120 92.1048 |  |  |  |  |  |  |  |  |  |
| OTU_992 | Hypocreales | 4 | 1 | 76.3180 | ASV_01429 76.3180 |  |  |  |  |  |  |  |  |  |

Table S2: Mixed-effects linear model of Kerguelen OTU abundance as a function of environmental variables. Fixed effects are reported with estimates, standard errors, degrees of freedom, t- and p-values. Random effects included published study, PCR primers, and sample type to account for study- and sampling-related biases. Only significant effects (p < 0.05) are reported. Abundance values were log-transformed and all environmental predictors were scaled. MAT: mean annual temperatures, SOC: soil organic carbon content.

| Predictor | Estimate | Standard error | df | t value | p value |
| --- | --- | --- | --- | --- | --- |
| Latitude² | 0.155 | 0.092 | 1172.40 | 1.68 | 0.033 |
| MAT | -0.462 | 0.073 | 1986.83 | -6.32 | <0.001 |
| pH | 0.273 | 0.049 | 3374.05 | 5.57 | <0.001 |
| SOC | 0.249 | 0.055 | 2629.69 | 4.52 | <0.001 |

Table S3: Ubiquity and and tundra specificity of the Kerguelen OTUs present in the GlobalFungi database. Ubiquity indicates the number of plant biomes (1 to 11) in which an OTU has been observed, while tundra specificity represents the proportion of sample points comprising the OTU of interest located in one of the tundra biomes. OTUs are ordered by the number of biomes they occupy.

| OTU | Ubiquity | Tundra specificity | Category |
| --- | --- | --- | --- |
| OTU_221 | 11 | 0.37051793 | Non-plant-associated |
| OTU_476 | 10 | 0.42325581 | Non-plant-associated |
| OTU_758 | 10 | 0.28143713 | Non-plant-associated |
| OTU_575 | 10 | 0.24734982 | Non-plant-associated |
| OTU_117 | 10 | 0.21284404 | Non-plant-associated |
| OTU_31 | 10 | 0.21284404 | Non-plant-associated |
| OTU_427 | 10 | 0.21284404 | Non-plant-associated |
| OTU_650 | 10 | 0.1960784 | Plant-associated |
| OTU_121 | 9 | 0.50000000 | Non-plant-associated |
| OTU_289 | 9 | 0.50000000 | Non-plant-associated |
| OTU_1243 | 9 | 0.41196013 | Non-plant-associated |
| OTU_76 | 9 | 0.41196013 | Non-plant-associated |
| OTU_246 | 9 | 0.32057416 | Non-plant-associated |
| OTU_47 | 9 | 0.22932331 | Non-plant-associated |
| OTU_1959 | 9 | 0.16129032 | Non-plant-associated |
| OTU_1678 | 9 | 0.05333333 | Non-plant-associated |
| OTU_912 | 8 | 0.47474747 | Non-plant-associated |
| OTU_50 | 8 | 0.40425532 | Non-plant-associated |
| OTU_1695 | 8 | 0.22596154 | Non-plant-associated |
| OTU_1735 | 8 | 0.15533981 | Non-plant-associated |
| OTU_712 | 8 | 0.05769231 | Non-plant-associated |
| OTU_268 | 7 | 0.74500000 | Non-plant-associated |
| OTU_507 | 7 | 0.44827586 | Non-plant-associated |
| OTU_1071 | 7 | 0.29411765 | Non-plant-associated |
| OTU_578 | 7 | 0.06707317 | Non-plant-associated |
| OTU_122 | 6 | 0.74666667 | Non-plant-associated |
| OTU_3188 | 6 | 0.66666667 | Non-plant-associated |
| OTU_643 | 6 | 0.64285714 | Non-plant-associated |
| OTU_826 | 6 | 0.54765751 | Non-plant-associated |
| OTU_236 | 6 | 0.40243902 | Non-plant-associated |
| OTU_392 | 6 | 0.30555556 | Non-plant-associated |
| OTU_711 | 6 | 0.24444444 | Non-plant-associated |
| OTU_700 | 6 | 0.20000000 | Non-plant-associated |
| OTU_1888 | 6 | 0.18918919 | Non-plant-associated |
| OTU_1754 | 6 | 0.15420561 | Non-plant-associated |
| OTU_450 | 6 | 0.14285714 | Non-plant-associated |
| OTU_2712 | 6 | 0.05555556 | Non-plant-associated |
| OTU_1124 | 6 | 0.04166667 | Non-plant-associated |
| OTU_293 | 6 | 0.00000000 | Non-plant-associated |
| OTU_3398 | 6 | 0.00000000 | Non-plant-associated |
| OTU_169 | 5 | 0.85156250 | Non-plant-associated |
| OTU_469 | 5 | 0.75862069 | Non-plant-associated |
| OTU_459 | 5 | 0.69175627 | Non-plant-associated |
| OTU_230 | 5 | 0.67500000 | Non-plant-associated |
| OTU_143 | 5 | 0.62500000 | Non-plant-associated |
| OTU_120 | 5 | 0.60000000 | Non-plant-associated |
| OTU_1332 | 5 | 0.50000000 | Non-plant-associated |
| OTU_240 | 5 | 0.41666667 | Non-plant-associated |
| OTU_901 | 5 | 0.32258065 | Non-plant-associated |
| OTU_48 | 5 | 0.25974026 | Non-plant-associated |
| OTU_1898 | 5 | 0.14285714 | Non-plant-associated |
| OTU_3273 | 5 | 0.14285714 | Non-plant-associated |
| OTU_2136 | 5 | 0.13793103 | Non-plant-associated |
| OTU_1538 | 5 | 0.10542169 | Non-plant-associated |
| OTU_2507 | 5 | 0.00000000 | Non-plant-associated |
| OTU_1444 | 4 | 0.88524590 | Non-plant-associated |
| OTU_642 | 4 | 0.87500000 | Non-plant-associated |
| OTU_2140 | 4 | 0.84210526 | Non-plant-associated |
| OTU_1081 | 4 | 0.82051282 | Non-plant-associated |
| OTU_856 | 4 | 0.81818182 | Non-plant-associated |
| OTU_2919 | 4 | 0.75000000 | Non-plant-associated |
| OTU_1897 | 4 | 0.71428571 | Non-plant-associated |
| OTU_2356 | 4 | 0.60000000 | Non-plant-associated |
| OTU_420 | 4 | 0.60000000 | Non-plant-associated |
| OTU_689 | 4 | 0.60000000 | Non-plant-associated |
| OTU_2381 | 4 | 0.57894737 | Non-plant-associated |
| OTU_485 | 4 | 0.44444444 | Non-plant-associated |
| OTU_224 | 4 | 0.33333333 | Non-plant-associated |
| OTU_928 | 4 | 0.29411765 | Non-plant-associated |
| OTU_744 | 4 | 0.27272727 | Non-plant-associated |
| OTU_994 | 4 | 0.26666667 | Non-plant-associated |
| OTU_237 | 4 | 0.22222222 | Non-plant-associated |
| OTU_260 | 4 | 0.14398200 | Non-plant-associated |
| OTU_372 | 4 | 0.00000000 | Non-plant-associated |
| OTU_174 | 4 | 0.7285714 | Plant-associated |
| OTU_34 | 4 | 0.1785714 | Plant-associated |
| OTU_400 | 3 | 0.91025641 | Non-plant-associated |
| OTU_580 | 3 | 0.90909091 | Non-plant-associated |
| OTU_1197 | 3 | 0.87500000 | Non-plant-associated |
| OTU_1407 | 3 | 0.87500000 | Non-plant-associated |
| OTU_1569 | 3 | 0.80000000 | Non-plant-associated |
| OTU_1263 | 3 | 0.78571429 | Non-plant-associated |
| OTU_361 | 3 | 0.71428571 | Non-plant-associated |
| OTU_254 | 3 | 0.57142857 | Non-plant-associated |
| OTU_45 | 3 | 0.57142857 | Non-plant-associated |
| OTU_1782 | 3 | 0.52500000 | Non-plant-associated |
| OTU_2073 | 3 | 0.50000000 | Non-plant-associated |
| OTU_567 | 3 | 0.50000000 | Non-plant-associated |
| OTU_2142 | 3 | 0.42857143 | Non-plant-associated |
| OTU_836 | 3 | 0.37500000 | Non-plant-associated |
| OTU_1840 | 3 | 0.25000000 | Non-plant-associated |
| OTU_2402 | 3 | 0.20000000 | Non-plant-associated |
| OTU_570 | 3 | 0.20000000 | Non-plant-associated |
| OTU_853 | 3 | 0.12500000 | Non-plant-associated |
| OTU_1136 | 3 | 0.00000000 | Non-plant-associated |
| OTU_154 | 3 | 0.00000000 | Non-plant-associated |
| OTU_2599 | 3 | 0.00000000 | Non-plant-associated |
| OTU_564 | 3 | 0.00000000 | Non-plant-associated |
| OTU_658 | 3 | 0.00000000 | Non-plant-associated |
| OTU_134 | 3 | 0.9354839 | Plant-associated |
| OTU_144 | 3 | 0.7500000 | Plant-associated |
| OTU_2854 | 2 | 1.00000000 | Non-plant-associated |
| OTU_467 | 2 | 1.00000000 | Non-plant-associated |
| OTU_77 | 2 | 1.00000000 | Non-plant-associated |
| OTU_840 | 2 | 1.00000000 | Non-plant-associated |
| OTU_899 | 2 | 1.00000000 | Non-plant-associated |
| OTU_745 | 2 | 0.92857143 | Non-plant-associated |
| OTU_621 | 2 | 0.91666667 | Non-plant-associated |
| OTU_1837 | 2 | 0.75000000 | Non-plant-associated |
| OTU_515 | 2 | 0.75000000 | Non-plant-associated |
| OTU_579 | 2 | 0.75000000 | Non-plant-associated |
| OTU_1140 | 2 | 0.66666667 | Non-plant-associated |
| OTU_1288 | 2 | 0.66666667 | Non-plant-associated |
| OTU_1892 | 2 | 0.66666667 | Non-plant-associated |
| OTU_552 | 2 | 0.66666667 | Non-plant-associated |
| OTU_1356 | 2 | 0.60000000 | Non-plant-associated |
| OTU_857 | 2 | 0.60000000 | Non-plant-associated |
| OTU_1058 | 2 | 0.50000000 | Non-plant-associated |
| OTU_142 | 2 | 0.50000000 | Non-plant-associated |
| OTU_1420 | 2 | 0.50000000 | Non-plant-associated |
| OTU_1596 | 2 | 0.50000000 | Non-plant-associated |
| OTU_1819 | 2 | 0.50000000 | Non-plant-associated |
| OTU_2284 | 2 | 0.50000000 | Non-plant-associated |
| OTU_280 | 2 | 0.50000000 | Non-plant-associated |
| OTU_305 | 2 | 0.50000000 | Non-plant-associated |
| OTU_444 | 2 | 0.50000000 | Non-plant-associated |
| OTU_46 | 2 | 0.50000000 | Non-plant-associated |
| OTU_568 | 2 | 0.50000000 | Non-plant-associated |
| OTU_67 | 2 | 0.50000000 | Non-plant-associated |
| OTU_710 | 2 | 0.50000000 | Non-plant-associated |
| OTU_759 | 2 | 0.50000000 | Non-plant-associated |
| OTU_941 | 2 | 0.50000000 | Non-plant-associated |
| OTU_783 | 2 | 0.46153846 | Non-plant-associated |
| OTU_1437 | 2 | 0.33333333 | Non-plant-associated |
| OTU_1657 | 2 | 0.25000000 | Non-plant-associated |
| OTU_536 | 2 | 0.25000000 | Non-plant-associated |
| OTU_197 | 2 | 0.22222222 | Non-plant-associated |
| OTU_1287 | 2 | 0.11111111 | Non-plant-associated |
| OTU_2186 | 2 | 0.09523810 | Non-plant-associated |
| OTU_1826 | 2 | 0.04761905 | Non-plant-associated |
| OTU_1396 | 2 | 0.00000000 | Non-plant-associated |
| OTU_1481 | 2 | 0.00000000 | Non-plant-associated |
| OTU_1687 | 2 | 0.00000000 | Non-plant-associated |
| OTU_173 | 2 | 0.00000000 | Non-plant-associated |
| OTU_178 | 2 | 0.00000000 | Non-plant-associated |
| OTU_1799 | 2 | 0.00000000 | Non-plant-associated |
| OTU_1831 | 2 | 0.00000000 | Non-plant-associated |
| OTU_324 | 2 | 0.00000000 | Non-plant-associated |
| OTU_398 | 2 | 0.00000000 | Non-plant-associated |
| OTU_439 | 2 | 0.00000000 | Non-plant-associated |
| OTU_558 | 2 | 0.00000000 | Non-plant-associated |
| OTU_631 | 2 | 0.00000000 | Non-plant-associated |
| OTU_822 | 2 | 0.00000000 | Non-plant-associated |
| OTU_870 | 2 | 0.00000000 | Non-plant-associated |
| OTU_98 | 2 | 0.00000000 | Non-plant-associated |
| OTU_83 | 2 | 0.8333333 | Plant-associated |
| OTU_90 | 2 | 0.7500000 | Plant-associated |
| OTU_664 | 2 | 0.6666667 | Plant-associated |
| OTU_771 | 2 | 0.6666667 | Plant-associated |
| OTU_682 | 2 | 0.5000000 | Plant-associated |
| OTU_384 | 2 | 0.4000000 | Plant-associated |
| OTU_325 | 2 | 0.3333333 | Plant-associated |
| OTU_175 | 2 | 0.0000000 | Plant-associated |
| OTU_203 | 2 | 0.0000000 | Plant-associated |
| OTU_957 | 2 | 0.0000000 | Plant-associated |
| OTU_1006 | 1 | 1.00000000 | Non-plant-associated |
| OTU_1025 | 1 | 1.00000000 | Non-plant-associated |
| OTU_1113 | 1 | 1.00000000 | Non-plant-associated |
| OTU_1194 | 1 | 1.00000000 | Non-plant-associated |
| OTU_1212 | 1 | 1.00000000 | Non-plant-associated |
| OTU_1348 | 1 | 1.00000000 | Non-plant-associated |
| OTU_137 | 1 | 1.00000000 | Non-plant-associated |
| OTU_140 | 1 | 1.00000000 | Non-plant-associated |
| OTU_1598 | 1 | 1.00000000 | Non-plant-associated |
| OTU_1733 | 1 | 1.00000000 | Non-plant-associated |
| OTU_184 | 1 | 1.00000000 | Non-plant-associated |
| OTU_1874 | 1 | 1.00000000 | Non-plant-associated |
| OTU_1889 | 1 | 1.00000000 | Non-plant-associated |
| OTU_1895 | 1 | 1.00000000 | Non-plant-associated |
| OTU_191 | 1 | 1.00000000 | Non-plant-associated |
| OTU_1914 | 1 | 1.00000000 | Non-plant-associated |
| OTU_2180 | 1 | 1.00000000 | Non-plant-associated |
| OTU_241 | 1 | 1.00000000 | Non-plant-associated |
| OTU_255 | 1 | 1.00000000 | Non-plant-associated |
| OTU_265 | 1 | 1.00000000 | Non-plant-associated |
| OTU_282 | 1 | 1.00000000 | Non-plant-associated |
| OTU_310 | 1 | 1.00000000 | Non-plant-associated |
| OTU_328 | 1 | 1.00000000 | Non-plant-associated |
| OTU_390 | 1 | 1.00000000 | Non-plant-associated |
| OTU_468 | 1 | 1.00000000 | Non-plant-associated |
| OTU_478 | 1 | 1.00000000 | Non-plant-associated |
| OTU_538 | 1 | 1.00000000 | Non-plant-associated |
| OTU_562 | 1 | 1.00000000 | Non-plant-associated |
| OTU_592 | 1 | 1.00000000 | Non-plant-associated |
| OTU_623 | 1 | 1.00000000 | Non-plant-associated |
| OTU_64 | 1 | 1.00000000 | Non-plant-associated |
| OTU_671 | 1 | 1.00000000 | Non-plant-associated |
| OTU_674 | 1 | 1.00000000 | Non-plant-associated |
| OTU_695 | 1 | 1.00000000 | Non-plant-associated |
| OTU_702 | 1 | 1.00000000 | Non-plant-associated |
| OTU_833 | 1 | 1.00000000 | Non-plant-associated |
| OTU_864 | 1 | 1.00000000 | Non-plant-associated |
| OTU_1048 | 1 | 0.00000000 | Non-plant-associated |
| OTU_1060 | 1 | 0.00000000 | Non-plant-associated |
| OTU_1096 | 1 | 0.00000000 | Non-plant-associated |
| OTU_1128 | 1 | 0.00000000 | Non-plant-associated |
| OTU_1154 | 1 | 0.00000000 | Non-plant-associated |
| OTU_1180 | 1 | 0.00000000 | Non-plant-associated |
| OTU_1201 | 1 | 0.00000000 | Non-plant-associated |
| OTU_1213 | 1 | 0.00000000 | Non-plant-associated |
| OTU_1214 | 1 | 0.00000000 | Non-plant-associated |
| OTU_1230 | 1 | 0.00000000 | Non-plant-associated |
| OTU_1242 | 1 | 0.00000000 | Non-plant-associated |
| OTU_1306 | 1 | 0.00000000 | Non-plant-associated |
| OTU_1337 | 1 | 0.00000000 | Non-plant-associated |
| OTU_1404 | 1 | 0.00000000 | Non-plant-associated |
| OTU_1415 | 1 | 0.00000000 | Non-plant-associated |
| OTU_1427 | 1 | 0.00000000 | Non-plant-associated |
| OTU_1439 | 1 | 0.00000000 | Non-plant-associated |
| OTU_1446 | 1 | 0.00000000 | Non-plant-associated |
| OTU_1486 | 1 | 0.00000000 | Non-plant-associated |
| OTU_155 | 1 | 0.00000000 | Non-plant-associated |
| OTU_1557 | 1 | 0.00000000 | Non-plant-associated |
| OTU_1573 | 1 | 0.00000000 | Non-plant-associated |
| OTU_1608 | 1 | 0.00000000 | Non-plant-associated |
| OTU_1651 | 1 | 0.00000000 | Non-plant-associated |
| OTU_1665 | 1 | 0.00000000 | Non-plant-associated |
| OTU_167 | 1 | 0.00000000 | Non-plant-associated |
| OTU_1673 | 1 | 0.00000000 | Non-plant-associated |
| OTU_1697 | 1 | 0.00000000 | Non-plant-associated |
| OTU_1727 | 1 | 0.00000000 | Non-plant-associated |
| OTU_1736 | 1 | 0.00000000 | Non-plant-associated |
| OTU_1835 | 1 | 0.00000000 | Non-plant-associated |
| OTU_1843 | 1 | 0.00000000 | Non-plant-associated |
| OTU_1845 | 1 | 0.00000000 | Non-plant-associated |
| OTU_1861 | 1 | 0.00000000 | Non-plant-associated |
| OTU_1886 | 1 | 0.00000000 | Non-plant-associated |
| OTU_1904 | 1 | 0.00000000 | Non-plant-associated |
| OTU_1998 | 1 | 0.00000000 | Non-plant-associated |
| OTU_2010 | 1 | 0.00000000 | Non-plant-associated |
| OTU_2090 | 1 | 0.00000000 | Non-plant-associated |
| OTU_2101 | 1 | 0.00000000 | Non-plant-associated |
| OTU_215 | 1 | 0.00000000 | Non-plant-associated |
| OTU_2163 | 1 | 0.00000000 | Non-plant-associated |
| OTU_2202 | 1 | 0.00000000 | Non-plant-associated |
| OTU_2208 | 1 | 0.00000000 | Non-plant-associated |
| OTU_2246 | 1 | 0.00000000 | Non-plant-associated |
| OTU_227 | 1 | 0.00000000 | Non-plant-associated |
| OTU_2275 | 1 | 0.00000000 | Non-plant-associated |
| OTU_2294 | 1 | 0.00000000 | Non-plant-associated |
| OTU_23 | 1 | 0.00000000 | Non-plant-associated |
| OTU_235 | 1 | 0.00000000 | Non-plant-associated |
| OTU_2358 | 1 | 0.00000000 | Non-plant-associated |
| OTU_239 | 1 | 0.00000000 | Non-plant-associated |
| OTU_2637 | 1 | 0.00000000 | Non-plant-associated |
| OTU_2703 | 1 | 0.00000000 | Non-plant-associated |
| OTU_291 | 1 | 0.00000000 | Non-plant-associated |
| OTU_299 | 1 | 0.00000000 | Non-plant-associated |
| OTU_327 | 1 | 0.00000000 | Non-plant-associated |
| OTU_351 | 1 | 0.00000000 | Non-plant-associated |
| OTU_356 | 1 | 0.00000000 | Non-plant-associated |
| OTU_357 | 1 | 0.00000000 | Non-plant-associated |
| OTU_3630 | 1 | 0.00000000 | Non-plant-associated |
| OTU_385 | 1 | 0.00000000 | Non-plant-associated |
| OTU_386 | 1 | 0.00000000 | Non-plant-associated |
| OTU_409 | 1 | 0.00000000 | Non-plant-associated |
| OTU_410 | 1 | 0.00000000 | Non-plant-associated |
| OTU_412 | 1 | 0.00000000 | Non-plant-associated |
| OTU_414 | 1 | 0.00000000 | Non-plant-associated |
| OTU_417 | 1 | 0.00000000 | Non-plant-associated |
| OTU_425 | 1 | 0.00000000 | Non-plant-associated |
| OTU_435 | 1 | 0.00000000 | Non-plant-associated |
| OTU_445 | 1 | 0.00000000 | Non-plant-associated |
| OTU_455 | 1 | 0.00000000 | Non-plant-associated |
| OTU_461 | 1 | 0.00000000 | Non-plant-associated |
| OTU_463 | 1 | 0.00000000 | Non-plant-associated |
| OTU_464 | 1 | 0.00000000 | Non-plant-associated |
| OTU_474 | 1 | 0.00000000 | Non-plant-associated |
| OTU_540 | 1 | 0.00000000 | Non-plant-associated |
| OTU_554 | 1 | 0.00000000 | Non-plant-associated |
| OTU_561 | 1 | 0.00000000 | Non-plant-associated |
| OTU_574 | 1 | 0.00000000 | Non-plant-associated |
| OTU_617 | 1 | 0.00000000 | Non-plant-associated |
| OTU_62 | 1 | 0.00000000 | Non-plant-associated |
| OTU_632 | 1 | 0.00000000 | Non-plant-associated |
| OTU_678 | 1 | 0.00000000 | Non-plant-associated |
| OTU_705 | 1 | 0.00000000 | Non-plant-associated |
| OTU_718 | 1 | 0.00000000 | Non-plant-associated |
| OTU_737 | 1 | 0.00000000 | Non-plant-associated |
| OTU_738 | 1 | 0.00000000 | Non-plant-associated |
| OTU_773 | 1 | 0.00000000 | Non-plant-associated |
| OTU_799 | 1 | 0.00000000 | Non-plant-associated |
| OTU_808 | 1 | 0.00000000 | Non-plant-associated |
| OTU_861 | 1 | 0.00000000 | Non-plant-associated |
| OTU_892 | 1 | 0.00000000 | Non-plant-associated |
| OTU_917 | 1 | 0.00000000 | Non-plant-associated |
| OTU_918 | 1 | 0.00000000 | Non-plant-associated |
| OTU_919 | 1 | 0.00000000 | Non-plant-associated |
| OTU_937 | 1 | 0.00000000 | Non-plant-associated |
| OTU_96 | 1 | 0.00000000 | Non-plant-associated |
| OTU_992 | 1 | 0.00000000 | Non-plant-associated |
| OTU_196 | 1 | 1.0000000 | Plant-associated |
| OTU_202 | 1 | 1.0000000 | Plant-associated |
| OTU_208 | 1 | 1.0000000 | Plant-associated |
| OTU_297 | 1 | 1.0000000 | Plant-associated |
| OTU_378 | 1 | 1.0000000 | Plant-associated |
| OTU_509 | 1 | 1.0000000 | Plant-associated |
| OTU_63 | 1 | 1.0000000 | Plant-associated |
| OTU_1038 | 1 | 0.0000000 | Plant-associated |
| OTU_1119 | 1 | 0.0000000 | Plant-associated |
| OTU_112 | 1 | 0.0000000 | Plant-associated |
| OTU_14 | 1 | 0.0000000 | Plant-associated |
| OTU_157 | 1 | 0.0000000 | Plant-associated |
| OTU_186 | 1 | 0.0000000 | Plant-associated |
| OTU_210 | 1 | 0.0000000 | Plant-associated |
| OTU_2115 | 1 | 0.0000000 | Plant-associated |
| OTU_261 | 1 | 0.0000000 | Plant-associated |
| OTU_273 | 1 | 0.0000000 | Plant-associated |
| OTU_3 | 1 | 0.0000000 | Plant-associated |
| OTU_341 | 1 | 0.0000000 | Plant-associated |
| OTU_388 | 1 | 0.0000000 | Plant-associated |
| OTU_422 | 1 | 0.0000000 | Plant-associated |
| OTU_44 | 1 | 0.0000000 | Plant-associated |
| OTU_487 | 1 | 0.0000000 | Plant-associated |
| OTU_491 | 1 | 0.0000000 | Plant-associated |
| OTU_534 | 1 | 0.0000000 | Plant-associated |
| OTU_58 | 1 | 0.0000000 | Plant-associated |
| OTU_607 | 1 | 0.0000000 | Plant-associated |
| OTU_629 | 1 | 0.0000000 | Plant-associated |
| OTU_71 | 1 | 0.0000000 | Plant-associated |
| OTU_81 | 1 | 0.0000000 | Plant-associated |
| OTU_9 | 1 | 0.0000000 | Plant-associated |
| OTU_939 | 1 | 0.0000000 | Plant-associated |
| OTU_965 | 1 | 0.0000000 | Plant-associated |

**REFERENCES**

1. Loidi J, Navarro-Sánchez G, Vynokurov D. Climatic definitions of the world’s terrestrial biomes. *Veg Classif Surv* 2022;**3**:231–271. https://doi.org/10.3897/VCS.86102

2. Ercole E, Adamo M, Lumini E, Fusconi A, Mucciarelli M. Alpine constructed wetlands: A metagenomic analysis reveals microbial complementary structure. *Sci Total Environ* 2022;**822**:153640. https://doi.org/10.1016/j.scitotenv.2022.153640

3. Egidi E, Delgado-Baquerizo M, Plett JM, Wang J, Eldridge DJ, Bardgett RD, et al. A few Ascomycota taxa dominate soil fungal communities worldwide. *Nat Commun* 2019;**10**:2369. https://doi.org/10.1038/s41467-019-10373-z
